# Investigation of D76N β_2_-Microglobulin Using Protein Footprinting and Structural Mass Spectrometry

**DOI:** 10.1021/jasms.0c00438

**Published:** 2021-02-15

**Authors:** Owen Cornwell, James R. Ault, Nicholas J. Bond, Sheena E. Radford, Alison E. Ashcroft

**Affiliations:** † Biopharmaceuticals R & D, AstraZeneca, Granta Park, Cambridge CB21 6GP, U.K.; ‡ Astbury Centre for Structural Molecular Biology & School of Molecular and Cellular Biology, University of Leeds, Leeds LS2 9JT, U.K.

**Keywords:** β_2_m, HDX, FPOP, amyloid, D76N, protein conformation, structural mass spectrometry

## Abstract

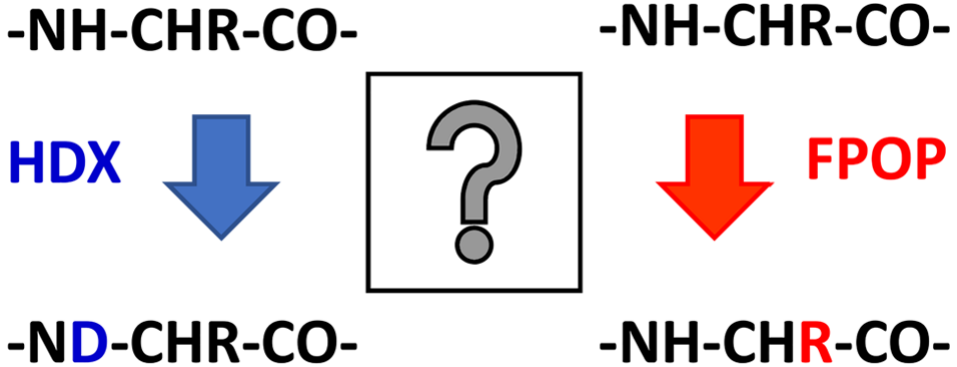

NMR studies and X-ray crystallography have shown that the structures of the 99-residue amyloidogenic protein β_2_-microglobulin (β_2_m) and its more aggregation-prone variant, D76N, are indistinguishable, and hence, the reason for the striking difference in their aggregation propensities remains elusive. Here, we have employed two protein footprinting methods, hydrogen–deuterium exchange (HDX) and fast photochemical oxidation of proteins (FPOP), in conjunction with ion mobility-mass spectrometry, to probe the differences in conformational dynamics of the two proteins. Using HDX-MS, a clear difference in HDX protection is observed between these two proteins in the E-F loop (residues 70–77) which contains the D76N substitution, with a significantly higher deuterium uptake being observed in the variant protein. Conversely, following FPOP-MS only minimal differences in the level of oxidation between the two proteins are observed in the E–F loop region, suggesting only modest side-chain movements in that area. Together the HDX-MS and FPOP-MS data suggest that a tangible perturbation to the hydrogen-bonding network in the E–F loop has taken place in the D76N variant and furthermore illustrate the benefit of using multiple complementary footprinting methods to address subtle, but possibly biologically important, differences between highly similar proteins.

## Introduction

The study of protein structure and dynamics is essential to the development of effective therapeutics targeted against illnesses associated with protein misfolding and aggregation, a category which includes prominent amyloid diseases such as Alzheimer’s, Parkinson’s, and type 2 diabetes mellitus.^1,2^ Protein footprinting coupled with mass spectrometry has become an increasingly useful strategy for the analysis of protein structure and dynamics^3,4^ particularly for larger proteins or more heterogeneous samples typically not amenable to high-resolution structural techniques such as NMR, X-ray crystallography, or cryo-EM.

Two protein footprinting methods frequently used in conjunction with mass spectrometry are hydrogen–deuterium exchange (HDX)^5^ and fast photochemical oxidation of proteins (FPOP).^6^ In a typical HDX-MS experiment, the protein analyte is incubated for varying lengths of time in a deuterated buffer solution, conditions under which solvent-accessible and labile hydrogen atoms on the protein (i.e., O–H, N–H, and S–H groups not involved in hydrogen bonds^7^) exchange, over time, with the deuterium in the buffer, increasing the mass of these regions of the protein molecule. The HDX reaction is quenched at low temperature and pH to minimize further forward exchange, as well as any back exchange, before the protein analyte is digested, typically with acid proteases (usually pepsin^8^) and the resulting peptides subjected to LC–MS/MS analysis. Mass increases are quantified using a weighted average mass of the peptide isotope distribution, often presented as an “uptake plot” for each peptide, showing mass increase relative to the undeuterated peptide, versus time.^8^ These data can be compared between different proteins or between different states of the same protein (e.g., with and without a ligand) to identify regions of differential deuterium uptake and thus likely regions of structural/dynamical changes in the protein. Although rapid back exchange usually limits HDX to the study of backbone amide hydrogens,^9^ this method has proved to be a key tool in the field of structural MS to study protein conformational changes as well as protein–protein and protein–ligand interactions.^5,7^

FPOP is a complementary footprinting method to HDX, in which hydroxyl radicals liberated from UV flash photolysis of hydrogen peroxide are used to covalently label solvent accessible amino acid side chains resulting in a variety of oxidative mass additions.^10,11^ The oxidized sample is typically proteolyzed and subjected to LC–MS/MS analysis, whereby the resulting amino acid modifications, most commonly +16 Da mass additions (overall addition of an oxygen atom) although many others are possible (e.g., + 32 Da (addition of two oxygen atoms) and +14 Da (methylene to carbonyl conversion)),^12^ can be readily identified at residue level resolution.^8,13^ These data are quantified using a label-free, area-under-the-curve integration strategy comparing the LC chromatograms of the unmodified and modified versions of each peptide to determine a percentage of modification for each oxidized species.^8,13−15^ Similar to HDX, these data can be compared between protein states, where changes in the extent of modification between states are indicative of changes in protein structure or dynamics. Despite being a relatively new addition to the structural MS toolbox, FPOP has occupied a useful niche in the covalent footprinting analysis of proteins and has thus far shown promise for a wide variety of analytes including membrane proteins,^16−18^ biopharmaceuticals,^14,19−22^ and even *in vivo* protein structural analysis.^23^

FPOP and HDX exhibit several characteristics which make them valuable companions when used as complementary methods. For example, FPOP labeling is generally considered to occur on the microsecond–millisecond time scale^24,25^ (depending on solution conditions^26^), whereas HDX more commonly labels on the seconds–hours time scale,^8^ although millisecond labeling times can be achieved using rapid mixing strategies.^27,28^ Owing to the covalent nature of the FPOP label, residue level resolution can be readily achieved using standard ergodic MS/MS fragmentation techniques, such as collision-induced dissociation (CID), without the concern of label scrambling. However, due to the significant differences in the reactivity of different amino acids to hydroxyl radical labeling, and subsequent peptide signal depletion from each new oxidized species, FPOP data are often sparse, frequently only providing data on approximately one in five residues.^8,29^ This is in contrast to HDX experiments, where high sequence coverages are common, but peptide level resolution is the normative level of analysis, owing to the challenges surrounding minimizing deuterium scrambling for residue level quantification, including the necessary use of nonergodic fragmentation methods such as electron transfer dissociation (ETD).^30^ As a result, the balanced shortfalls and advantages of both HDX and FPOP make these two approaches complementary for the analysis of protein structure and dynamics and have been used in combination for an increasingly wide variety of studies including conformational analysis of amyloidogenic proteins,^8^ peptide therapeutics,^31^ Fc binding interactions,^32^ and numerous epitope mapping studies.^14,20−22^

**Figure 1:**
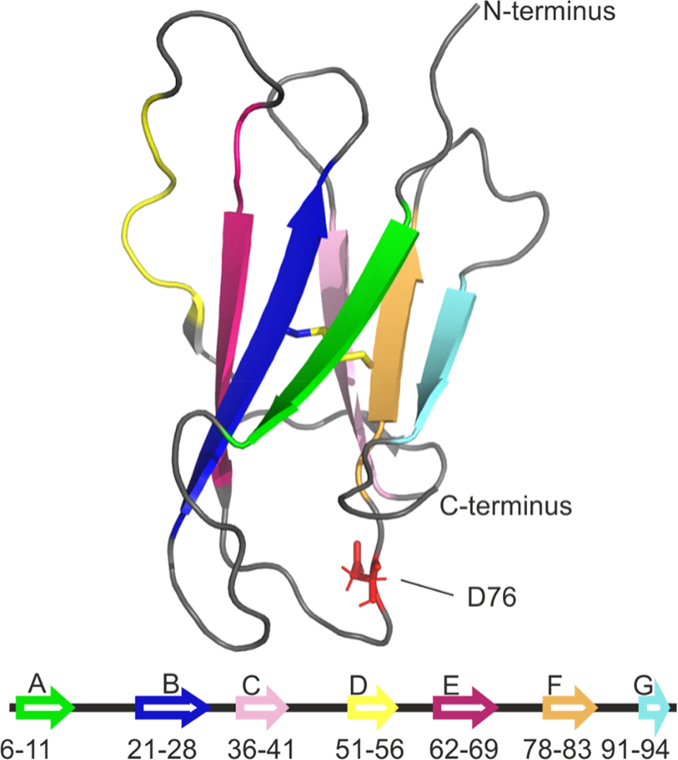
Structure of wild-type β_2_-microglobulin. β-Strands A–G are colored as in the lower diagram. The single disulfide bond between residues 25 and 80 (strands B and F) is shown as yellow sticks. The D76 side chain in the E–F loop is highlighted and shown as red sticks. PDB: 2XKS.^42^

Here, we utilize FPOP-MS and HDX-MS, as well as native IMS-MS, to compare wild-type β_2_-microglobulin (β_2_m), a small, 99 residue immunoglobulin (Ig) domain protein, with its highly aggregation-prone variant D76N, which has an Asp-Asn substitution at position 76.^33^ Wild-type β_2_m has a typical Ig “β-sandwich” structure consisting of seven antiparallel β-strands organized into two separate β-sheets which are connected by a single disulfide bond (Figure 1). *In vivo*, β_2_m forms the light chain component of the major histocompatibility complex (MHC-1).^34−36^ Aggregation of wild-type β_2_m into amyloid fibrils localized to the osteoarticular tissues (i.e., bones and joints) occurs in long-term hemodialysis patients with elevated levels of the protein in a condition known as dialysis-related amyloidosis (DRA).^37,38^ Although a precise mechanism of *in vivo* β_2_m aggregation in DRA is yet to be elucidated, β_2_m amyloidogenesis is believed to occur via a highly aggregation-prone folding intermediate known as the I_T_ state, containing a non-native *trans* peptide bond between residues His31 and Pro32^39^ which is thought to aggregate on the collagen surfaces in the joints.^40,41^

First reported in 2012, D76N is a naturally occurring, highly aggregation-prone variant of β_2_m with the Asp-Asn substitution at position 76 situated within the loop region linking the E and F β-strands of the protein (Figure 1).^33^ In a distinct disease pathology to that of wild-type β_2_m amyloidosis in DRA, individuals heterozygous for the genetic mutation causing D76N showed systemic amyloidosis which was not localized to the bones and joints but rather to the internal organs in which the amyloid deposits contain only the D76N variant.^33^ Importantly, and in contrast to the wild-type protein, the D76N variant is observed to be highly amyloidogenic *in vitro* at neutral pH in the absence of accessory molecules, conditions under which the wild-type protein does not form amyloid.^43,44^ Mutagenesis studies have shown that no other D–N amino acid substitution in β_2_m yields any notable increase in aggregation propensity,^43^ whereas several D76 substitutions (D76H, D76E, and D76A) do increase the propensity of the protein to form amyloid under the same experimental conditions as D76N,^45^ indicating that the position, rather than the specific nature of the amino acid substitution, is the important factor in mediating aggregation propensity.

Surprisingly, despite the stark increase in its aggregation propensity, the D76N variant has thus far proven to be strikingly similar to the wild-type protein using a variety of structural and biophysical techniques. For example, the crystal structure of D76N, and indeed those of other D76 variants for which crystal structures have been solved,^45^ are indistinguishable from that of the wild-type protein.^45^ Additionally, the MHC-1 complex containing D76N has been shown to have a similar structure, dissociation patterns, and stability compared to its wild-type β_2_m counterpart.^46^ The folding pathway of the D76N variant was found to be similar to that of the wild-type protein, and although some experiments have suggested that D76N has an increased proportion of the aggregation-prone I_T_ state folding intermediate at equilibrium,^47^ in line with other aggregation-prone variants of β_2_m,^39,48^ subsequent CD and NMR analyses have shown no significant difference to the wild-type β_2_m.^44^ Similarly, several *in silico* studies have suggested that the conformation, rather than the population, of the I_T_ state in D76N is distinct to that of the wild-type protein by comparatively increased disordering of the termini^49^ or the D-strand.^50^ Again, however, NMR analysis has shown the conformations of the D76N and wild-type β_2_m I_T_ state to be similar.^44^

In the face of these marked structural similarities, we utilize structural mass spectrometry, in the form of HDX-MS, FPOP-MS, and native IMS-MS, to compare the structure and dynamic properties of wild-type and D76N β_2_m and to determine if these methods can reveal differences between the two proteins which may rationalize the radical increase in aggregation propensity of the D76N variant.

## Methods

### Protein Preparation

Protein samples were expressed recombinantly and purified as described previously.^51^

### Native ESI-IMS-MS

Samples prepared for native MS were buffer exchanged twice into 150 mM ammonium acetate (pH 7.4) using 7k MWCO Zeba spin desalting columns (Thermo Scientific, Hemel Hempstead, UK). The final sample was diluted to a concentration of 10 μM, calculated based on the absorbance at 280 nm and the Beer–Lambert law, using an extinction coefficient of 20065 M^–1^ cm^–1^ for both wild-type and D76N β_2_m. Samples were then loaded into borosilicate glass capillaries pulled in-house (Sutter Instrument Company, Novato, CA) and coated with palladium using a sputter coater (Polaron SC7620, Quorum Technologies Ltd., Kent, UK).

Spectra were acquired using a Synapt G1 HDMS (Waters Corp., Wilmslow, UK) in positive ESI mode. MS and IMS settings were as follows: cone voltage: 70 V, backing pressure: 2.1 mbar, T-wave velocity: 300 ms^–1^, IMS T-wave height: 4–10 V ramp (100% cycle). Data were analyzed using MassLynx v4.1 and Driftscope v3.0 software (Waters Corp., Wilmslow, UK).

### FPOP–LC–MS/MS

The experimental setup used in FPOP experiments was as described previously.^8,15,16^ Immediately prior to UV irradiation (<10 s), 1 μL of 5% *v/v* H_2_O_2_ was added to 100 μL of protein solution containing 10 μM of either wild-type or D76N β_2_m and 20 mM l-histidine as an amino acid scavenger in 10 mM potassium phosphate pH 6.2 to give a final H_2_O_2_ concentration of 0.05% v/v. The sample was then passed at a fixed flow rate of 20 μL min^–1^ through a fused silica capillary (i.d. 100 μm) and irradiated with UV light (beam width ∼3 mm) using a Compex 50 Pro KrF excimer laser (Coherent Inc., Ely, UK) operating at 248 nm with a firing frequency of 15 Hz and a pulse duration of 20 ns. Laser power was kept constant at 100 mJ. Based on laminar flow modeling of the sample flow through the capillary,^52^ these experimental parameters are such that >90% of the sample experiences either one or zero UV irradiation events. Further details of these calculations and the experimental setup can be found in ref (53). The outflow from the capillary was collected into an Eppendorf tube containing 20 μL of quench solution (100 mM l-methionine, 1 μM catalase in 10 mM potassium phosphate pH 6.2) and placed immediately on ice. Following UV irradiation and quenching, the single disulfide bond in β_2_m (and D76N) was reduced by incubation with 10 mM DTT for 1 h at 55 °C, shaking at 500 rpm. The resulting free thiols were alkylated by incubation with 55 mM iodoacetamide for 45 min, 20 °C at 500 rpm in the dark. A 1:50 w/w ratio of chymotrypsin:protein was then added, and the mixture was left for 18 h at 37 °C, shaking at 500 rpm, to digest the samples into peptides. The resulting chymotryptic peptides (1 μL at 0.5 μM peptide concentration) were injected onto a UPLC M-Class Acquity system equipped with a C18 column (75 μm × 150 mm, Waters, Ltd., Wilmslow, UK) and separated by a gradient elution of 1–50% v/v MeCN in H_2_O, over 60 min at 0.3 μL min^–1^, where both mobile phases contained 0.1% v/v formic acid (∼pH 2.7). Peptides eluting from the UPLC were analyzed using a Q-Exactive Plus Orbitrap mass spectrometer (ThermoFisher, Bremen, Germany) operating in DDA mode with the following acquisition parameters: TopN = 5, max injection time = 300 ms, dynamic exclusion = 3 s. Experiments were performed in triplicate.

Peptides were identified using PEAKS v8.5 software (Bioinformatics Solution, Inc., Waterloo, ON, Canada). Variable mass additions of +16, + 32, and +14 Da were searched to identify FPOP modifications. MS/MS data were manually curated to identify and assign genuine FPOP oxidations. Data were then quantified manually at the residue level using Xcalibur software (v4.0.27.19, ThermoFisher, Bremen, Germany) by integrating peaks in the extracted ion chromatograms (XICs) of each peptide ion, generated by extracting the *m*/*z* of the base peak of each peptide isotope distribution, for each charge state, for the modified and unmodified versions of each peptide using the following equation:
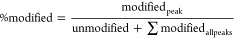


### HDX-MS

The HDX-MS setup used was as described previously.^8^ A 30 μL sample of protein stock solution containing 8 μM of either wild-type or D76N β_2_m in equilibration buffer (10 mM potassium phosphate, pH 6.2) was added to 135 μL of deuterated buffer (10 mM potassium phosphate, pD 6.2). This was incubated at 4 °C for 30, 60, 120, 1800, or 7200 s before 50 μL of the labeled solution was quenched by dilution into 100 μL of quench buffer (10 mM potassium phosphate, 2 M guanidine HCl, 200 mM tris(2-carboxyethyl)phosphine pH 2.2) at 1 °C, giving a final quench pH ∼ 2.5. A 50 μL sample of quenched sample (∼24 pmol) was passed through an immobilized ethylene-bridged hybrid (BEH) pepsin column (Waters, Ltd., Wilmslow, UK) at 20 °C at a flow rate of 500 μL min^–1^ before the resulting peptides were trapped using a VanGuard precolumn Acquity UPLC BEH C18 trap column (1.7 μm, 2.1 μm × 5 μm, Waters, Ltd., Wilmslow, UK). After valve switching, the resulting peptic peptides were transferred to a C18 column (75 μm × 150 mm, Waters Ltd., Wilmslow, UK) and separated by gradient elution of 0–40% MeCN (0.1% v/v formic acid) in H_2_O (0.3% v/v formic acid) over 7 min at 40 μL min^–1^. Peptides were analyzed using a Synapt G2Si mass spectrometer (Waters, Ltd., Wilmslow, UK) operating in DIA mode. Each time point, including t = 0, was replicated five times.

HDX data were processed using Protein Lynx Global Server (PLGS v3.0.2) and DynamX (v3.0.0) software supplied with the mass spectrometer. Criteria for confidently identified peptides were as follows: min intensity = 1000, min products per amino acid = 0.3, max sequence length = 25, max ppm error = 5, file threshold = 4/5 replicates. To visualize data and generate difference plots and structural heat maps, further data processing was carried out using PAVED v0.9.1,^8^ which was downloaded for free at https://biologicalsciences.leeds.ac.uk/downloads/download/28/software_download.

## Results and Discussion

### Analysis of WT β_2_m and D76N β_2_m Using Native IMS-MS

**Figure 2:**
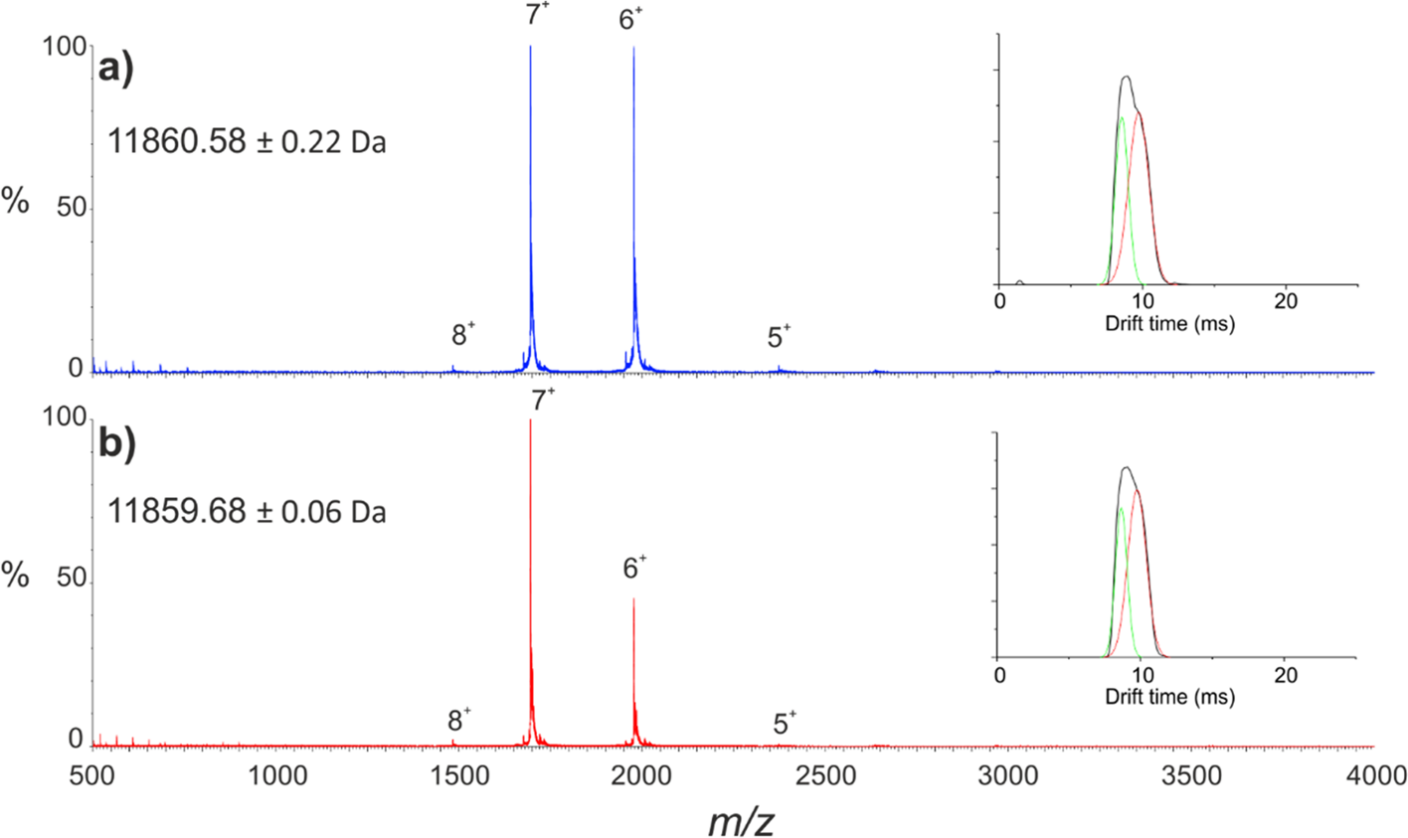
Native ESI-IMS-MS analysis of wild-type and D76N β_2_m. Native ESI-MS spectra for (a) wild-type β_2_m (blue) and (b) the D76N variant (red). Peaks are annotated with charge states. Calculated masses and standard deviations from each spectrum are shown in the top left-hand corner. Insets show the IMS arrival time distribution for the 6^+^ charge states (raw data are shown in black; fitted Gaussian curves in green and red).

**Figure 3:**
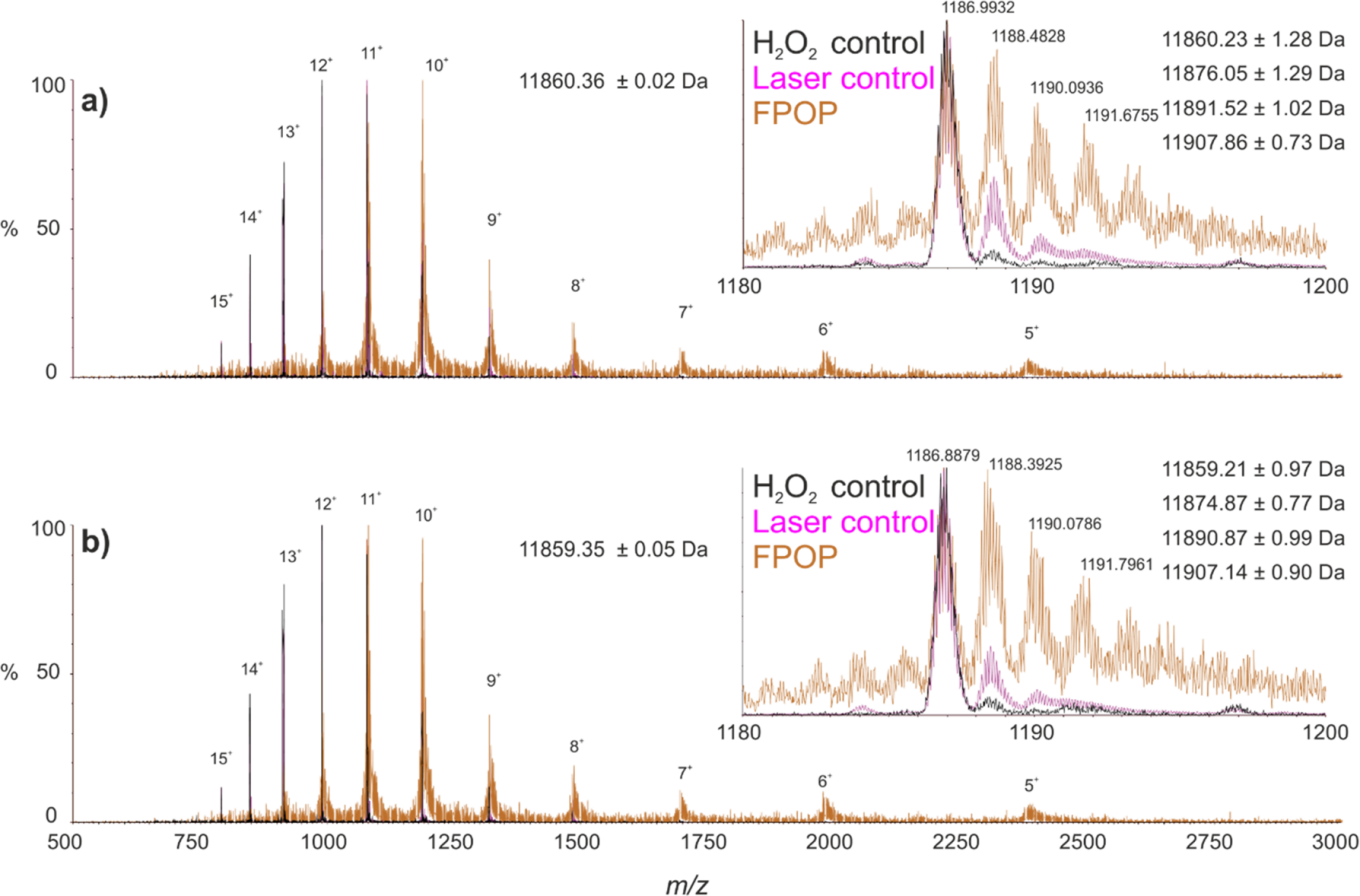
Denatured ESI-MS spectra of intact wild-type and D76N β_2_m following FPOP exposure. FPOP oxidized (orange) and non-FPOP control samples without irradiation (purple) and without irradiation or H_2_O_2_ exposure (black) are shown for (a) wild-type β_2_m and (b) the D76N variant. Peaks are annotated with charge state. Average mass and standard deviation for the control spectra without irradiation or H_2_O_2_ exposure are shown. Insets highlight the 10^+^ charge state ions from each spectrum. Calculated masses and standard deviations from each species observed in the FPOP oxidized spectra are shown in the top right-hand corner.

Wild-type and D76N β_2_m were first subjected to native ESI-IMS-MS analysis to determine whether any global structural changes could be observed. Samples of wild-type and D76N β_2_m were buffer exchanged independently into 150 mM ammonium acetate solution at neutral pH and diluted to a concentration of 10 μM, conditions under which neither protein aggregates *in vitro*. The resulting spectra are shown in Figure 2a,b for the wild-type protein and the D76N variant, respectively. Figure 2 indicates the mass accuracy is sufficient to highlight the 1 Da difference between the two variants, consistent with the intact, denatured spectra (see the control spectra in Figure 3). The charge-state distributions of the native wild-type β_2_m and D76N variant are similar, indicating a similar degree of folding under the conditions employed. This is further illustrated by inspection of the IMS data including the collision cross sections (CCSs; Supporting Information Table S1) and analysis of the arrival time distributions of each charge state, an example of which is shown for the 6+ charge state as an inset in each spectrum (Figure 2).

Together, the IMS data indicate the arrival time distributions of wild-type β_2_m and the D76N variant are highly comparable with similar drift times, peak widths, and relative intensities. These data further illustrate the similar structural characteristics of the wild-type and D76N variants and show that, to the resolution of these IMS experiments, these two proteins are largely indistinguishable.

### Probing Conformational Differences Using FPOP–LC–MS

To attain higher resolution structural data on wild-type and D76N β_2_m, both proteins were subjected to FPOP footprinting monitored by analysis of the intact proteins as well as by proteolysis followed by LC–MS/MS, to assess the extent of oxidative modification at both intact and residue level resolution. Intact, denatured mass spectra for samples subjected to FPOP oxidation, as well as two non-FPOP controls (samples exposed to hydrogen peroxide but not irradiated, and samples not exposed to either hydrogen peroxide or UV irradiation), are shown for wild-type and D76N β_2_m in Figures 3a,b, respectively. When subjected to FPOP, both proteins show significant modification relative to the control samples, largely in the form of +16 Da mass additions, although minor oxidation is also observed in the laser control samples (i.e., samples exposed to hydrogen peroxide but not UV irradiation) representing the background level of oxidation in the experiment. Similarly, common to the FPOP spectra is a significant decrease in the signal-to-noise ratio relative to that of the non-FPOP control spectra, owing to signal dilution caused by multiple oxidations, as well as a bias toward lower charge states, the latter most likely caused by the oxidation and subsequent resistance to protonation of histidine side-chains (β_2_m has six histidine residues).^13^ For both wild-type and D76N β_2_m, ∼65% of the total protein signal was observed to be oxidized under the conditions employed. However, due to the substantial differences in oxidation propensity of amino acids in FPOP experiments, it has been noted that changes in the degree of oxidation of certain residues can be masked by invariant or opposite-trending oxidation on residues more sensitive to hydroxyl radical labeling (e.g., methionine and tryptophan) making intact, or indeed peptide level, quantification challenging to interpret.^54^ To probe these data further, oxidized samples were digested with chymotrypsin and the resulting peptides subjected to LC–MS/MS analysis to identify and quantify modifications at the residue level.

**Figure 4:**
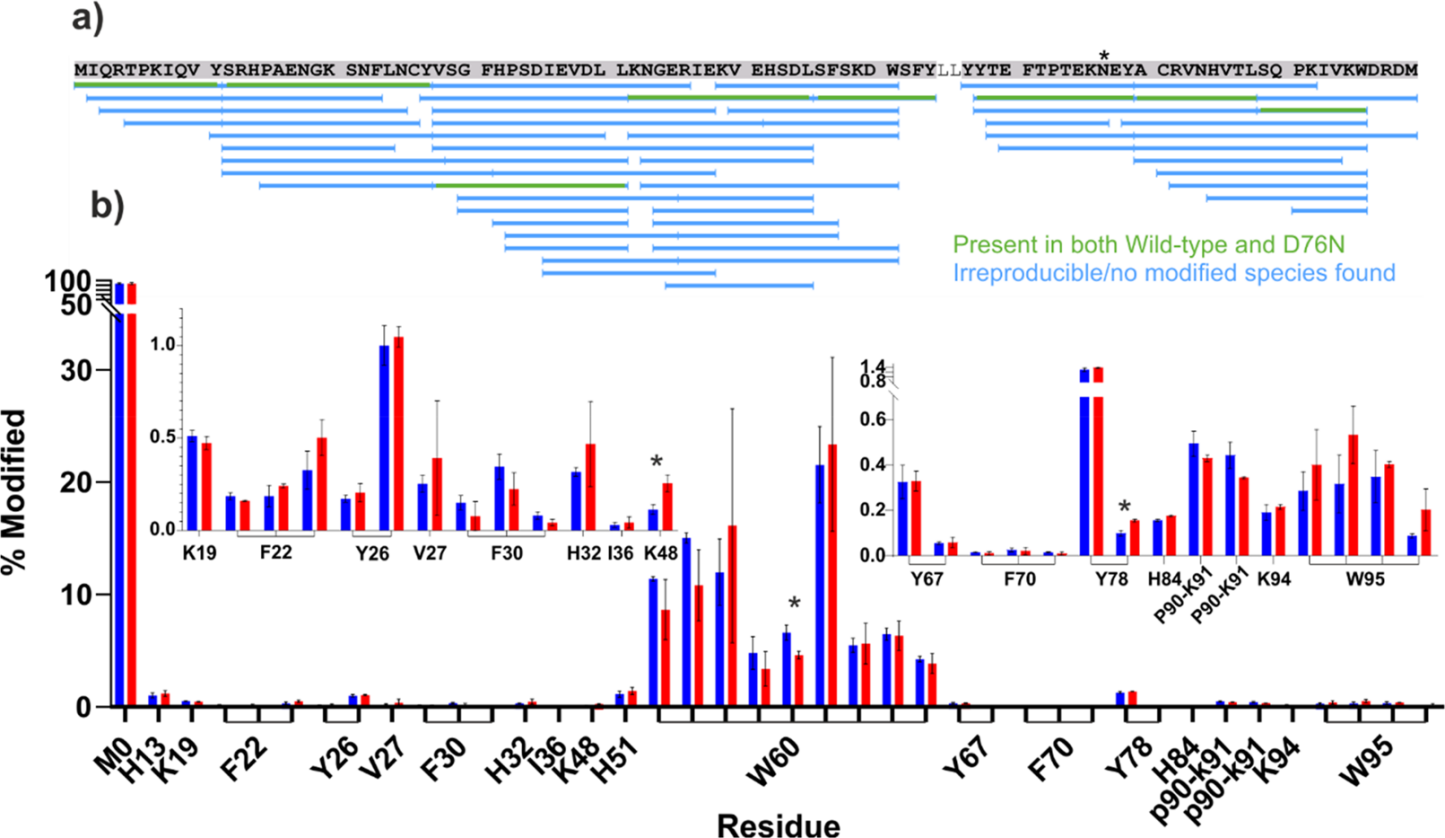
Residue level FPOP–LC–MS/MS analysis of wild-type and D76N β_2_m. (a) Sequence coverage map of the chymotrypsin digest of FPOP samples. Peptides used for quantification, which were present in all three replicates for both proteins, and which were observed to be present in both oxidized and unoxidized forms, are shown in green. The variant position, residue 76, is marked with a *. (b) Quantification of modified side chains for wild-type (blue) and D76N (red) β_2_m. Residues for which the quantification data are shown are annotated on the *x* axis. Instances where MS/MS data were insufficient to identify individual residues as the modified site show the range of residues to which the modification could be localized. Insets show zoomed *y* axis for regions K19–K48 (left) and Y67–W95 (right). Products identified as the same species at different retention times (i.e., positional isomers) are linked on the *x*-axis with brackets. Bars highlighted with an * indicate differences where *p* < 0.01. Error bars show standard deviation, *n* = 3.

Following proteolytic digestion and LC–MS/MS analysis, a total of eight chymotryptic peptides, covering 93% of the β_2_m sequence, were identified that were present in all FPOP replicates for both proteins and for which oxidized and unoxidized species could be identified reliably (highlighted in green in Figure 4a). Consistent with previous FPOP data from our laboratory on the wild-type protein under similar conditions,^8^ 40 different oxidized products were observed across 19 different amino acid side chains on the protein, all but two of which could be identified to single amino acid resolution. In agreement with previous data, multiple oxidation products were observed for individual Trp, Phe, and Tyr residues, consistent with the expected positional isomeric products resulting from hydroxyl radical attack at different positions on the aromatic rings of these side chains.^8^ For the laser control samples, LC–MS/MS analysis was able to reliably identify oxidations only on the N- and C-terminal methionine residues (Supporting Information Figure S1), which is not unexpected when taking into account the high solvent exposure and high reactivity of these side chains. Given the absence of the N-terminal methionine residue in the naturally occurring counterparts of these proteins, and the observation that C-terminal methionine oxidation was barely observed on doubly oxidized peptides, owing to the proximity of this residue to a highly reactive tryptophan side chain (Supporting Information Figure S1; Figure 4a), no correction for background oxidation was deemed necessary for these residues. Quantification data for all identified sites in FPOP oxidized samples, for both wild-type and D76N β_2_m, are shown in Figure 4b and listed in detail in Supporting Information Table S2.

After correcting for the increase in type 1 error associated with multiple comparisons, none of the modified sites observed met the *p* value threshold for statistical significance (Supporting Information Table S2), again highlighting the strong similarity between the two proteins. Three modified sites did show differences in the extent of modification between wild-type and D76N β_2_m where *p* < 0.01, namely: K48, a minor oxidation product of Y78, and one of the W60 oxidation products (Figure 4b). Conformational rearrangement and partial or complete burial of the normally solvent-exposed W60 side chain have been noted in several aggregation-prone variants of β_2_m,^55,56^ including the ΔN6 truncation variant,^8^ the latter which is generally considered to be a structural mimic of the aggregation-prone I_T_ state.^42,48^ However, although the W60 oxidation product indicating the most significant change in these experiments did show ∼30% relative reduction in oxidation compared with the wild-type protein, implying lower solvent accessibility, the remaining oxidation products of the W60 side chain in D76N are modified to a similar extent to those of the wild-type protein. Together with the observation that the oxidation profile for W60 in D76N is distinct to that observed for the ΔN6 variant in previous FPOP experiments under similar conditions,^8^ this most likely indicates that the structure of the D76N variant in this region is largely similar to that of the wild-type protein, with only minor conformational changes in this region.

**Figure 5:**
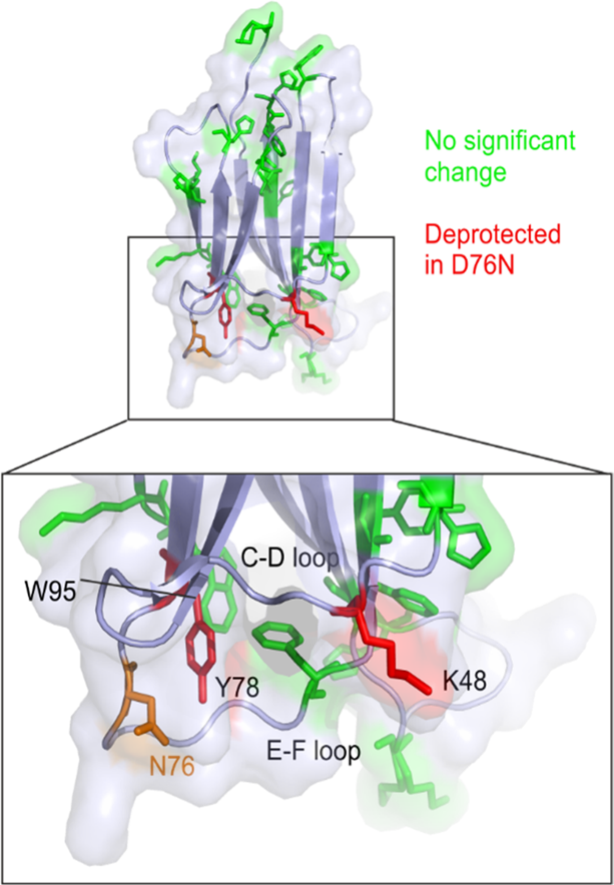
FPOP-oxidized amino acid side chains mapped onto the structure of the D76N variant of β_2_m. Side chains modified by FPOP are shown as sticks and highlighted in green. Residues K48 and Y78, both of which showed increased oxidation (*p* < 0.01) in D76N relative to the wild-type protein by FPOP, are shown as sticks and highlighted in red. The position of N76 is also highlighted (orange sticks). PDB: 4FXL.^33^

Interestingly, the two other sites which show the most significant (*p* < 0.01) changes in labeling between the two proteins, K48 (>2-fold increased labeling in D76N) and one (positional isomer) oxidation product of Y78 (∼1.6-fold increased labeling in D76N), are both located on the same side of the β-sandwich structure of the protein, in the vicinity of the D76N substitution site (Figure 5). As both positions show increased labeling relative to the wild-type protein, this may suggest an increased flexibility of this region of the D76N variant relative to that of the wild-type protein. Indeed, closer examination of W95, a residue spatially adjacent to the D76-containing E-F loop (Figure 5), reveals that although none exceeds the threshold for statistical significance, all four oxidation products identified for this residue show a trend of increased labeling in the D76N variant (Figure 4b). However, given that the major oxidation product of Y78, as well as several other oxidation sites nearby (K19, F70, Y67, and W95) show no significant change, the difference observed in the single Y78 positional isomer most likely reflects subtle fluctuations in solvent accessibility. Similarly, changes in peptide primary structure have been noted to modulate the oxidation profiles of residues in hydroxyl radical footprinting experiments^57^ and, although this effect has not been widely explored in the literature, the possibility that the D–N substitution itself may have an effect on the oxidation of nearby residues must be acknowledged.

However, given the overall similarity between wild-type β_2_m and the D76N variant observed using NMR, X-ray crystallography, and other techniques,^44,45^ the small differences observed in side-chain labeling close to the D76N substitution which have been identified in these FPOP experiments could be significant in contributing to its increased amyloidogenicity. To investigate further the structure of this region, wild-type and D76N β_2_m were next subjected to HDX-MS analysis.

### Probing Conformational Differences Using HDX-MS

**Figure 6:**
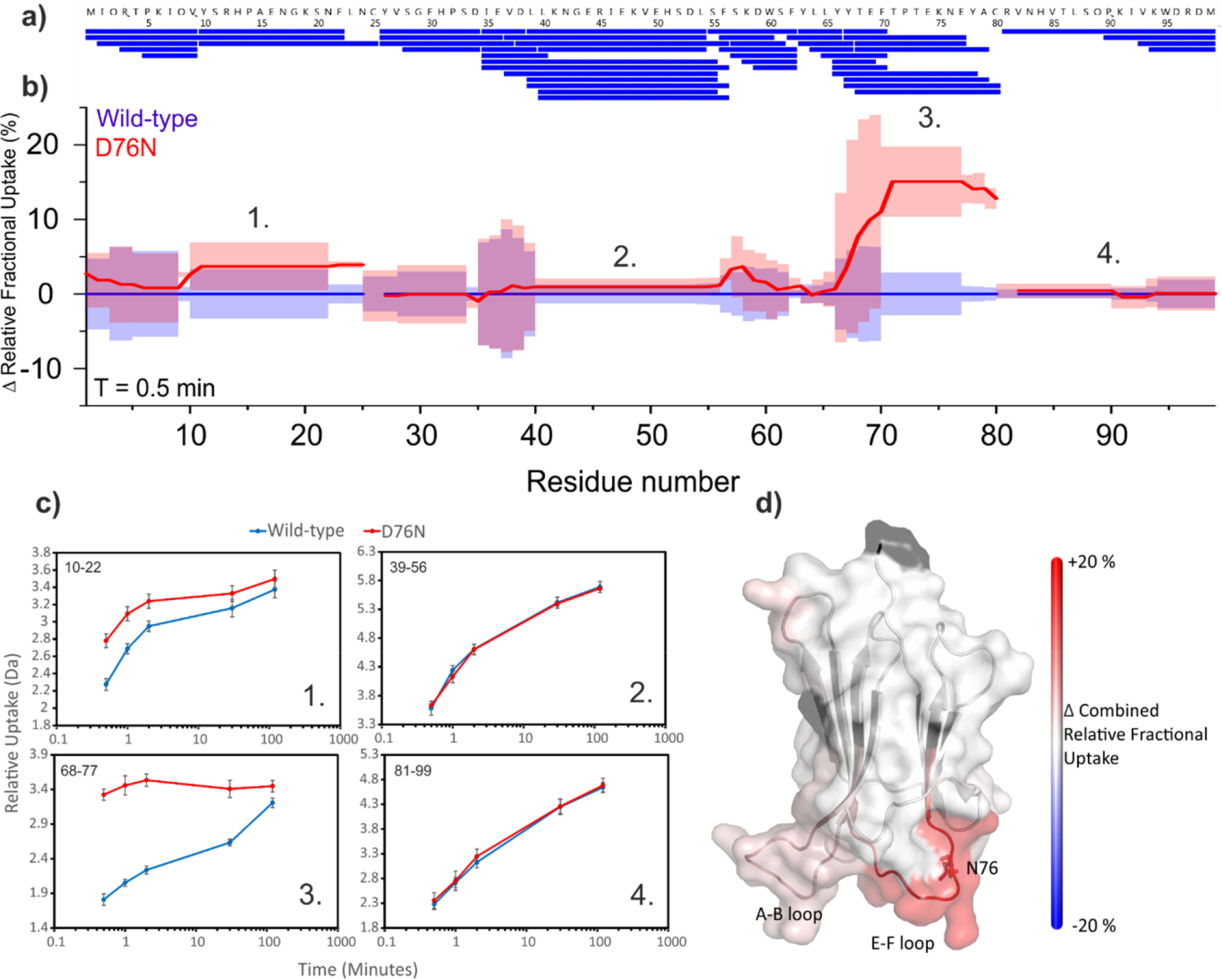
Differential HDX between wild-type and D76N β_2_m. (a) Sequence coverage map of the pepsin digest of HDX samples. (b) PAVED^8^ plot for the 30 s incubation time-point showing combined relative fractional uptake for wild-type β_2_m (blue, set to 0 for reference) and the D76N variant (red). Shaded regions show combined standard deviation, *n* = 5. (c) Uptake plots for four peptides covering different regions of β_2_m (numbered 1–4, positions in the protein sequence are as annotated in the PAVED plot in Figure 6b). Residues covered by each peptide are annotated in the top left-hand corner of each plot. Error bars show standard deviation, *n* = 5. (d) Structural heat map of differential hydrogen exchange. Black regions indicate the absence of data. Only statistically significant differences are shown (*p* < 0.05 based PAVED algorithm^8^) with an increase in deuterium incorporation for D76N β_2_m shown in red. PDB: 4FXL.^33^

The wild-type β_2_m and the D76N variant were next subjected to HDX-MS analysis to determine the effect, if any, of the D76N substitution on the solvent accessibility, stability and hydrogen bonding of backbone amides. Following online pepsin digestion, a total of 51 peptides covering 100% of the β_2_m sequence were identified (Figure 6a and Supporting Information Table S3) which were present reproducibly (five replicates) from both proteins and which met previously defined identification criteria^8^ (see the Methods). For the purpose of data analysis, the N-terminal residue of each peptide was discarded due to back-exchange issues,^58^ leaving two internal residues (in addition to the N-terminal methionine) without data: Y26 and R81. To ensure robust data analysis and simplicity of data presentation, the PAVED (positional averaging for visualizing exchange data) data analysis algorithm^8^ was used to process and present peptide uptake data. The resulting PAVED difference plot (Figure 6b) shows the change in relative fractional uptake (with wild-type β_2_m, as the reference state, being set to zero) versus residue number. A clear increase in deuterium uptake is observed in the D76N variant most notably in the E–F loop (residues 70–77) incorporating the D76N substitution (an ∼15% increase in relative fractional uptake, Figure 6c) with smaller increases in deuterium uptake also observed in the nearby A–B loop (residues 12–20, Figure 6d). Indeed, the uptake plots of all seven peptides which cover the D76N substitution site show substantial increases in deuterium uptake in the D76N variant relative to the wild-type protein (examples are shown in Figure 6c; uptake plots of all peptides are supplied in Supporting Information Figure S2). Although the D–N substitution itself increases the intrinsic deuterium exchange rate of this position slightly (approximately 2.5 fold under the conditions employed^59,60^), this effect is minimal within the context of the peptides’ overall uptake and, after correcting for back exchange, was not found to contribute significantly to the differences observed in uptake behavior between the two variants.^53^ Hence, the HDX data are strongly suggestive of a significant change in conformational dynamics of the E–F loop and also in the spatially adjacent A–B loop, resulting from the D76N substitution (Figure 6d). Interestingly, the most significant increases in uptake in these regions are observed at the earliest labeling time-points, whereas at longer deuterium incubation times much smaller differences in relative uptake are observed (Figure 6c and Supporting Information Figure S3). This suggests that the most rapidly exchanging amides near the D76N substitution site (the uptake of which constitute the bulk of the added deuterium at shorter incubation times) are the primary contributors to the difference in uptake observed between the two variants. It is also curious to note that, despite the significantly decreased global stability of the D76N variant relative to the wild-type protein,^47^ the deuterium uptake behavior in most other regions of the two proteins is similar. However, other HDX experiments performed in our laboratories on these two proteins under higher pH conditions do show global increased deuterium uptake at the longer incubation times,^53^ consistent with the decreased conformational stability of the D76N variant (data not shown). The lack of this observation in the experiments reported here is most likely due to the lower pH conditions employed and consequent decrease in intrinsic deuterium exchange rate.

Furthermore, minor variations in deuterium uptake behavior between the two variants are also observed close to the D-E loop (residues 57–60, Figure 6a), which may support the minor changes in FPOP oxidation observed for W60 in this region (Figure 4b). However, complex deuterium uptake behavior of overlapping peptides in this region makes a straightforward interpretation of the HDX data in this area challenging (Supporting Information Figure S2), and thus, a structural/dynamical difference between the two proteins in the D–E loop cannot be established with certainty from the available data.

**Figure 7:**
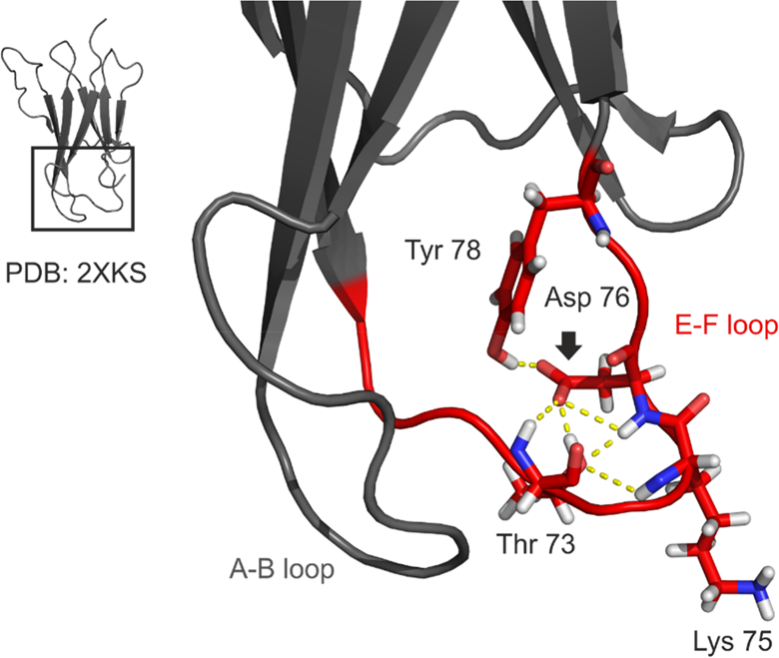
Hydrogen bonding in the E–F loop of wild-type β_2_m. Amino acid side-chains in the E–F loop (red) which are involved in hydrogen bonding are shown as sticks and colored by element: oxygen (red), nitrogen (blue), hydrogen (white). Polar contacts between atoms are shown as dashed yellow lines. D76 is annotated with a black arrow. The C terminus has been hidden for clarity. PDB: 2XKS.^42^

As noted in previous reports,^45^ D76 has been suggested as a critical node in the hydrogen bonding network in the E–F loop, a region observed to be rigid and well-ordered from NMR experiments.^42,45^ Indeed, polar contacts predicted from the NMR structure of wild-type β_2_m form numerous hydrogen bonds, including several backbone hydrogen bonds (changes in which are visible to the HDX experiments here) involving D76, T73 and K75 (Figure 7).^42^ Given the different hydrogen-bonding properties of the aspartic acid and asparagine side chains, it is reasonable to infer that the D76N substitution may cause some disruption to this interaction network, as suggested by others.^45,61^ Indeed, given the significant increases in deuterium uptake observed in the E–F loop of the D76N variant, the HDX data presented here strongly support this hypothesis, providing the first *in vitro* solution evidence that the D76N substitution frees or weakens polar interactions in this region. This explanation would also clarify the increases in deuterium uptake in the nearby A–B loop, as the increased flexibility of the E–F loop, brought about by weakened hydrogen bonding interactions, likely impacts the dynamics of this neighboring region also (Figure 7). Furthermore, this reasoning explains the observation of only minor fluctuations in the side-chain FPOP labeling in this region as, while the loss of hydrogen bonding would produce radical differences in deuterium uptake behavior, the subsequent increase in flexibility is not likely to drastically alter the solvent accessibility of side-chains which are already largely exposed to bulk solvent. However, as only 5 of the 20 residues which comprise the A–B and E–F loops label under the FPOP conditions employed, this discrepancy could feasibly be explained by the comparatively sparse data in this region using FPOP. That said, the observation that of the six side chains identified as undergoing FPOP labeling near the D76N substitution (K19, Y67, F70, Y76, K48 in the A–B and E–F loops and W95 close to the C-terminus), Y76, K48, and W95 all show oxidation products with a trend of increasing oxidation in the D76N variant, a plausible outcome of the likely increased average solvent accessibility from amplified loop flexibility, demonstrates the sensitivity of FPOP to even subtle changes in the solvent accessibility of amino acid side chains.

## Conclusions

The combined use of several structural mass spectrometric techniques for the comparison of wild-type β_2_m and its variant D76N demonstrates the power and utility of protein footprinting methods, as well as the advantages of utilizing orthogonal footprinting methods in combination to produce an understanding of protein structure and dynamics in molecular detail to a greater extent than the sum of either method alone.

The FPOP and HDX data presented provide the first solution phase evidence that the D76N substitution in the E–F loop of β_2_m disrupts the hydrogen-bonding interactions of this region, leading to a change in loop dynamics and an increase in flexibility of the E–F and A–B loops. Although how this relates to the aggregation pathway of D76N remains unclear, the observation by others that alternative D76 substitutions cause similar increases in aggregation propensity,^45^ changes which would likely similarly disrupt native E–F loop interactions, could highlight the hydrogen bonding network in this region as a key regulator of amyloid formation in β_2_m. The astounding structural similarity of wild-type and D76N β_2_m,^44−46^ which is also indicated in some of the nonfootprinting structural MS experiments presented here, demonstrates the significance of this finding as a step toward understanding and finding new strategies to mitigate the aggregation process.

## Supporting Information Available

FPOP-MS control peptide sequence map, FPOP-MS spectra and chromatogram comparing oxidized and unoxidized protein, FPOP-MS quantification data; HDX-MS raw data and uptake plots plus PAVED difference plots; IMS-MS estimated collision cross sections (PDF)
