# Supplementary material for: Investigation of D76N β2-Microglobulin Using Protein Footprinting and Structural Mass Spectrometry
Source: J Am Soc Mass Spectrom. 2021 Feb 15;32(7):1583–92. doi: 10.1021/jasms.0c00438 (PMC9282677; doi:10.1021/jasms.0c00438)
Supplement: Supplementary file 1 — js0c00438_si_001.pdf [file js0c00438_si_001.pdf]

Supporting Information

An investigation of D76N β_2-microglobulin using protein footprinting and structural mass spectrometry

Owen Cornwell[1], James R. Ault[2], Nicholas J. Bond[1], Sheena E. Radford[2*], Alison E. Ashcroft[2*]

1. Biopharmaceuticals R & D, AstraZeneca, Granta Park, Cambridge, CB21 6GP, UK.
2. Astbury Centre for Structural Molecular Biology & School of Molecular and Cellular Biology, University of Leeds, Leeds, LS2 9JT, UK.

[*] **Corresponding Authors:**
Alison E. Ashcroft, a.e.ashcroft@leeds.ac.uk
Sheena E. Radford, s.e.radford@leeds.ac.uk

Running title: D76N β_2m analysed using protein footprinting

Keywords: β_2m, HDX, FPOP, amyloid, D76N, protein conformation, protein dynamics, structural mass spectrometry

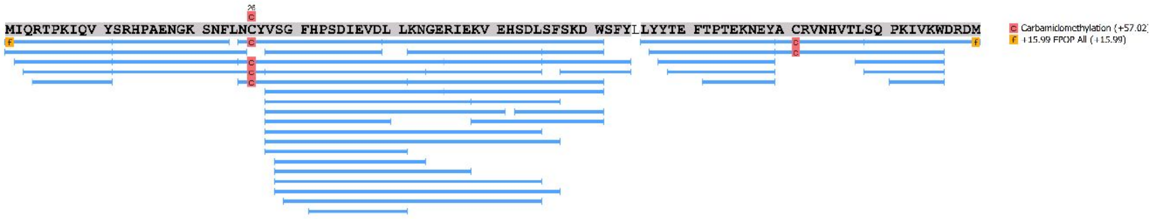

Figure S1. Example sequence coverage map of a non-FPOP control sample of D76N following chymotryptic digest. Only two modification sites were identified: those of the N- and C-terminal methionine residues.

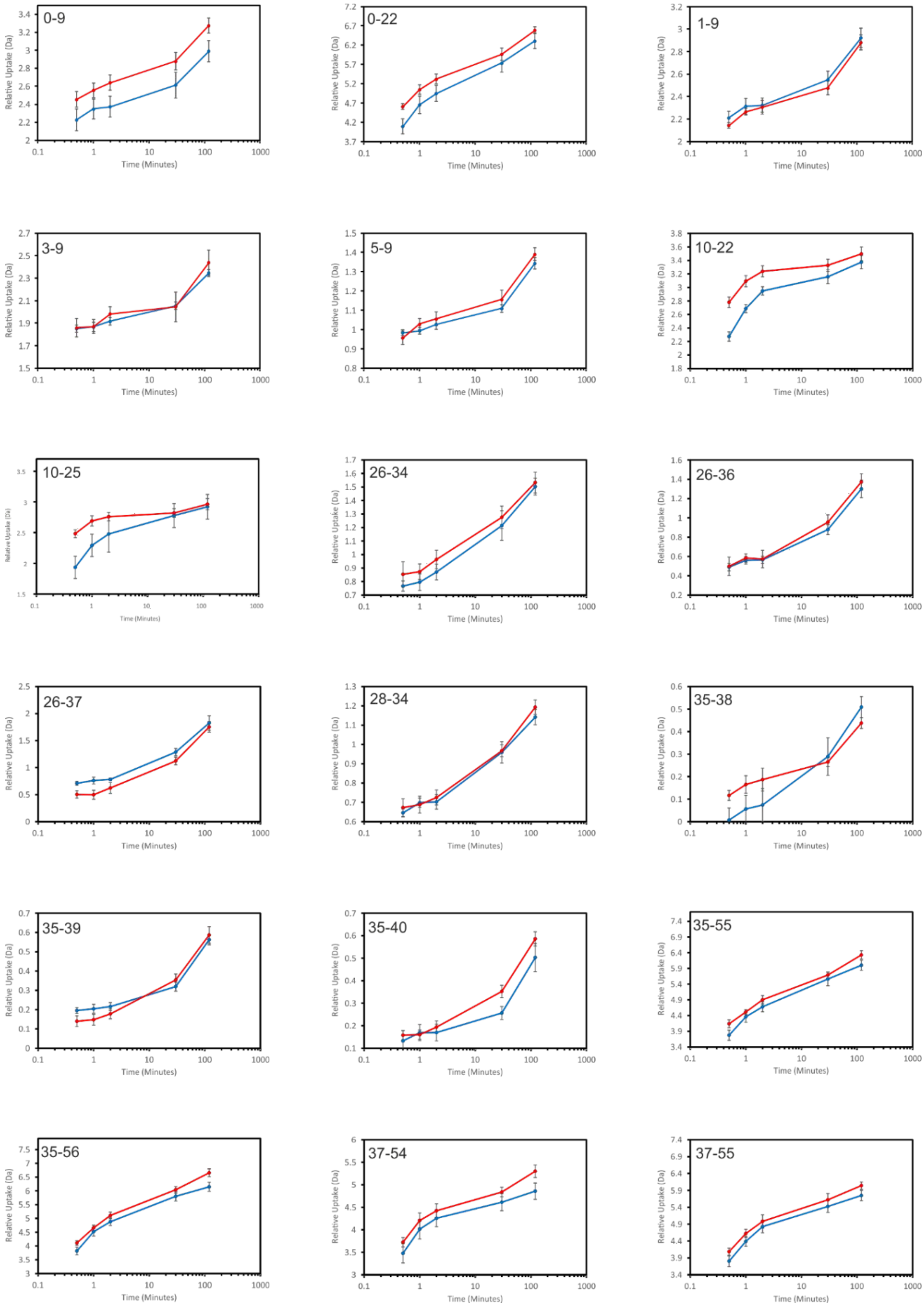
0-9
0-22
1-9
3-9
5-9
10-22
10-25
26-34
26-36
26-37
28-34
35-38
35-39
35-40
35-55
35-56
37-54
37-55
Relative Uptake (Da)
Time (Minutes)

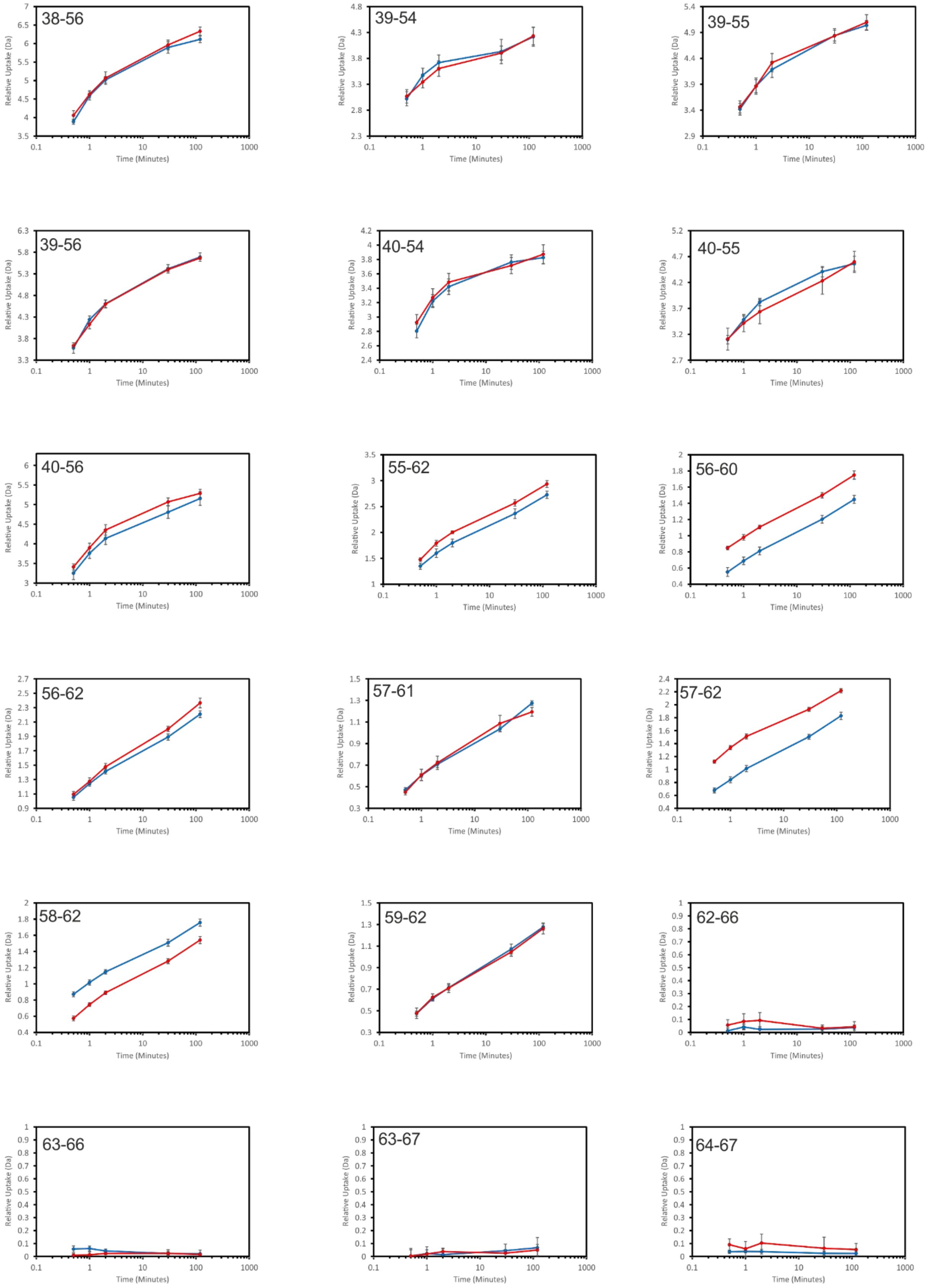
38-56
39-54
39-55
39-56
40-54
40-55
40-56
55-62
56-60
56-62
57-61
57-62
58-62
59-62
62-66
63-66
63-67
64-67
Relative Uptake (Da)
Time (Minutes)
0.1
1
10
100
1000

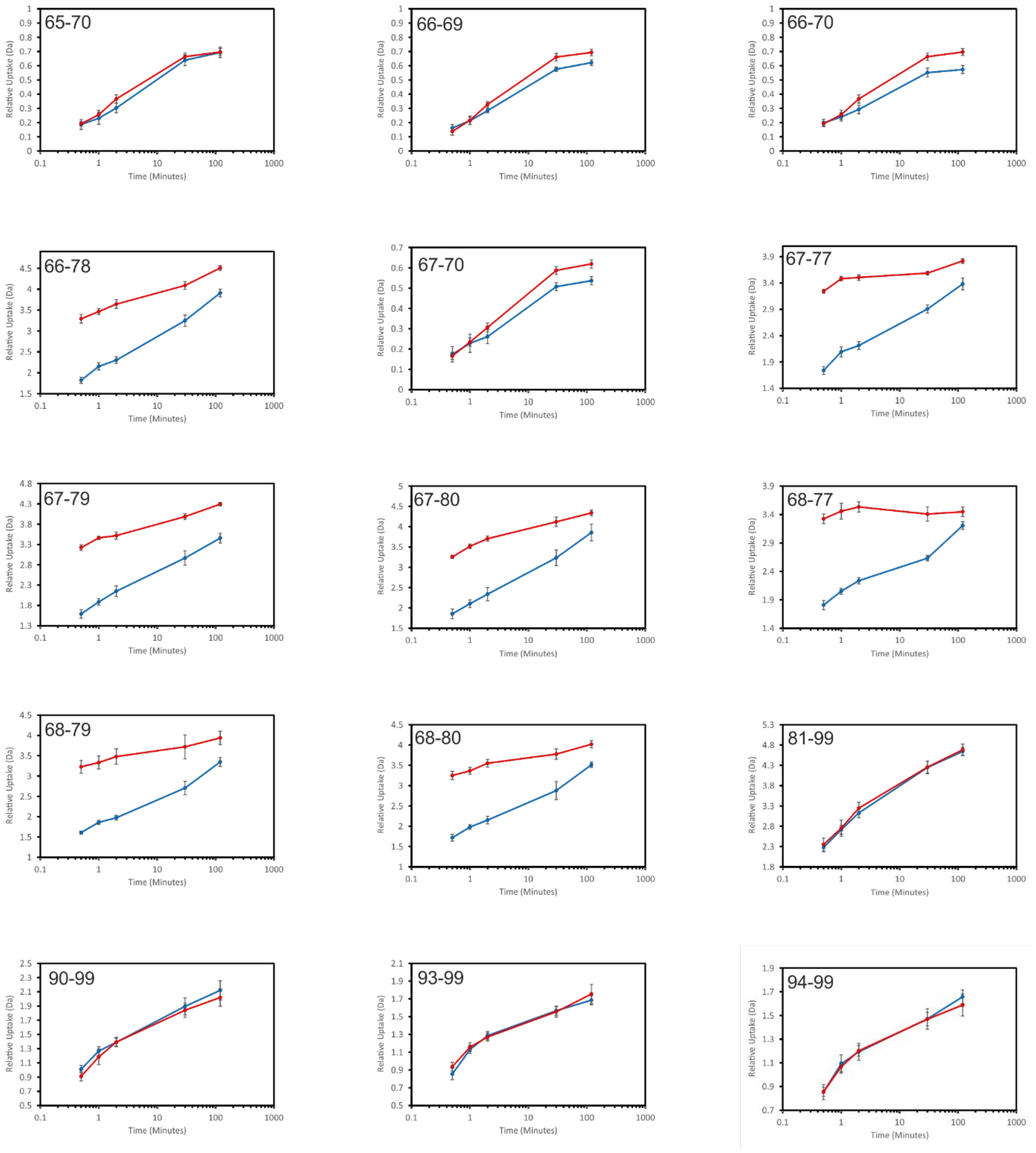

Figure S2. HDX-MS uptake plots from all 51 peptides identified for wild-type (blue) and D76N (red) β_2m. The residue numbers covered by the peptide are shown in the top left hand corner of each plot. Error bars show standard deviation, n=5.

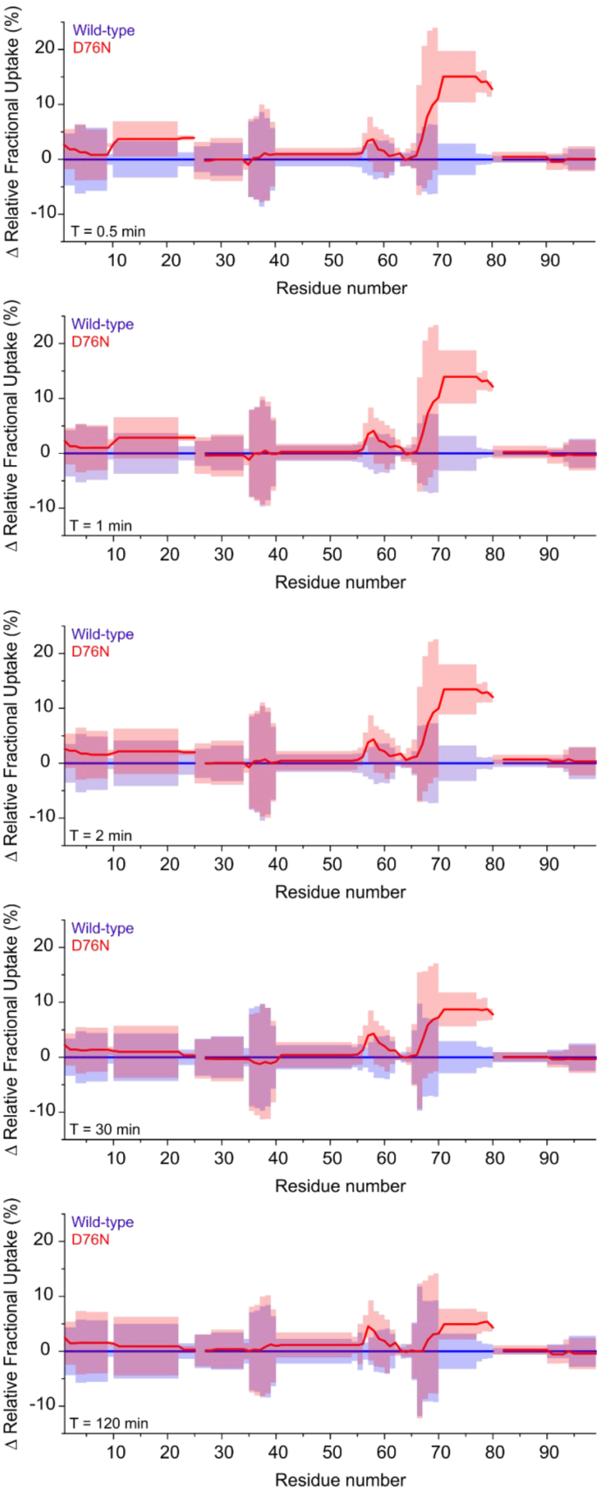

Figure S3. HDX-MS PAVED [1] difference plots for wild-type (blue) and D76N (red) β_2m over time. Shaded regions show combined standard deviation. The time-point for each plot is shown in the bottom left hand corner of each plot.

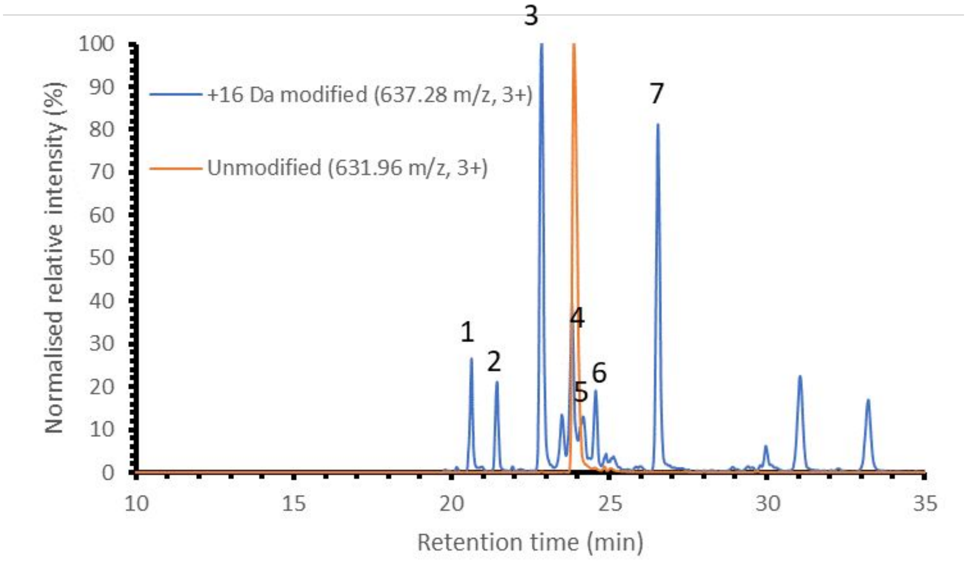

Figure S4. Example FPOP-MS extracted ion chromatograms for modified and unmodified versions of the chymotryptic peptide 11-26 (sequence: SRHPAENGKSNFLNCY) from β_2m. Modified peaks are annotated 1-7 as in the MS/MS data assigned to each peak in Figure S5.

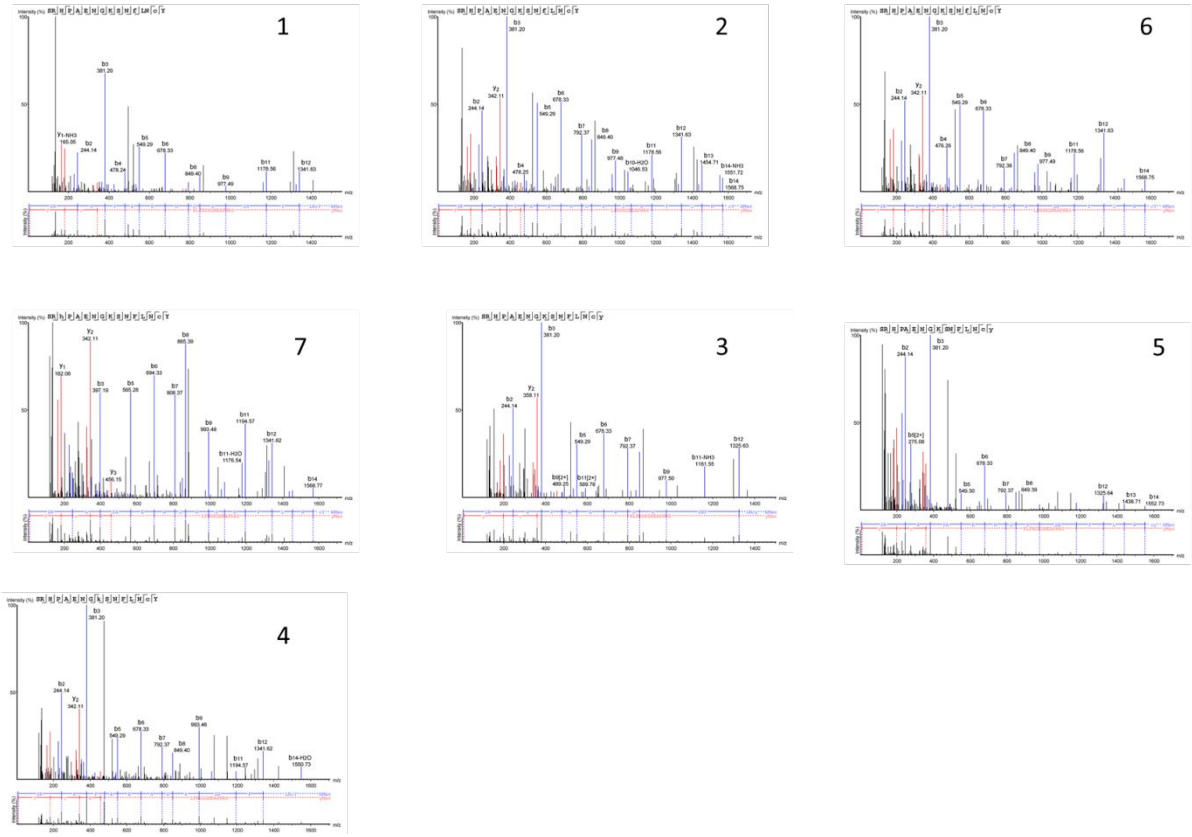

Figure S5. Example FPOP-MS/MS data for chymotryptic peptide 11-26 (sequence SRHPAENGKSNFLNCY) from β_2m. Spectra are annotated 1-7 as in Figure S4 which identifies the LC peaks from which these spectra are taken. Fragment ions are annotated with m/z and coloured blue and red for b and y ions, respectively

Table S1. Calculated ESI-IMS-MS collision cross-section (CCS) values for wild-type and D76N β_2-microglobulin. CCS values were calculated manually using native calibrant proteins from the Bush database of CCS values generated from drift cell measurements [2].

	**Rotationally averaged CCS (nm^2)**	
**Charge state**	**wild-type β_2-microglobulin**	**D76N β_2-microglobulin**
5+	12.43<br>13.30	12.34<br>13.16
6+	12.44<br>13.31	12.41<br>13.35
7+	15.45<br>15.51	15.45<br>16.04
8+	16.47	16.96

Table S2. Wild-type and D76N β_2m raw quantification data for FPOP-LC-MS experiments.

Unmodified		Modified					
**Peptide**	***m/z***	**Δmass (Da)**	***m/z***	**Wild-type (% Modified ± S.D)**	**D76N (% Modified ± S.D)**	**P value**	**Assignment**
**MIQRTPKIQVY (0-10)**	688.89 (2+)<br>464.93 (3+)	+16	696.88 (2+)<br>464.93 (3+)	93.43 ± 1.404	93.43 ± 1.939	0.9998	M0
**SRHPAENGKSNFLNCY (11-26)**	947.43 (2+)<br>631.96 (3+)<br>474.22 (4+)	+16	478.21 (4+)	1.04 ± 0.224	1.19 ± 0.262	0.493	His 13
		+16	478.21 (4+)<br>637.28 (3+)	0.51 ± 0.030	0.47 ± 0.034	0.201	Lys 19
		+16	478.21 (4+)<br>637.28 (3+)	0.19 ± 0.018	0.16 ± 0.003	0.046	Phe 22
		+16	478.21 (4+)<br>637.28 (3+)	0.19 ± 0.058	0.24 ± 0.010	0.215	Phe 22
		+16	478.21 (4+)<br>637.28 (3+)	0.33 ± 0.103	0.50 ± 0.096	0.105	Phe 22
		+16	478.21 (4+)<br>637.28 (3+)	0.17 ± 0.019	0.21 ± 0.049	0.258	Tyr 26
		+16	478.21 (4+)<br>637.28 (3+)	1.00 ± 0.108	1.05 ± 0.055	0.514	Tyr 26
**VSGFHPSDIEVDLL (27-40)**	764.38 (2+)	+ 16	772.38 (2+)	0.25 ± 0.045	0.39 ± 0.309	0.481	Val 27
		+16	772.38 (2+)	0.15 ± 0.038	0.08 ± 0.080	0.243	Phe 30
		+16	772.38 (2+)	0.35 ± 0.068	0.23 ± 0.088	0.135	Phe 30
		+16	772.38 (2+)	0.08 ± 0.019	0.04 ± 0.018	0.057	Phe 30
		+16	772.38 (2+)	0.32 ± 0.023	0.47 ± 0.230	0.324	His 32
		+16	772.38 (2+)	0.03 ± 0.013	0.04 ± 0.031	0.634	Ile 36
**KNGERIEKVEHSDL (41-54)**	827.43 (2+)<br>551.96 (3+)<br>414.22 (4)	+16	557.29 (3+)	0.11 ± 0.026	0.26 ± 0.044	0.007	Lys 48
		+16	557.29 (3+)	1.13 ± 0.279	1.43 ± 0.317	0.286	His 51
**SFSKDWSFY**	583.76 (2+)	+16	591.76 (2+)	11.40 ± 0.195	8.67 ± 2.681	0.153	Trp 60

**(55-63)**							
		+16	591.76 (2+)	15.05 ± 0.446	10.84 ± 3.159	0.084	Trp 60
		+16	591.76 (2+)	12.00 ± 2.952	16.14 ± 10.410	0.544	Trp 60
		+16	591.76 (2+)	4.81 ± 1.461	3.39 ± 1.531	0.310	Trp 60
		+16	591.76 (2+)	6.63 ± 0.677	4.61 ± 0.332	0.009	Trp 60
		+32	599.75 (2+)	21.54 ± 3.403	23.38 ± 7.746	0.726	Trp 60
		+32	599.75 (2+)	5.50 ± 0.620	5.63 ± 1.800	0.911	Trp 60
		+32	599.75 (2+)	6.47 ± 0.535	6.34 ± 1.312	0.881	Trp 60
		+32	599.75 (2+)	4.26 ± 0.232	3.86 ± 0.891	0.494	Trp 60
**YTEFTPTEKDEY (67-78)**	761.83 (2+) (WT) 761.34 (2+) (D76N)	+16	769.82 (2+) (WT) 769.34 (2+) (D76N)	0.33 ± 0.074	0.33 ± 0.044	1	Tyr 67
		+16	769.82 (2+)	0.06 ± 0.006	0.06 ± 0.022	1	Tyr 67
		+16	769.82 (2+)	0.02 ± 0.001	0.01 ± 0.008	0.098	Phe 70
		+16	769.82 (2+)	0.03 ± 0.008	0.02 ± 0.015	0.366	Phe 70
		+16	769.82 (2+)	0.01 ± 0.003	0.01 ± 0.007	1	Phe 70
		+16	769.82 (2+)	1.29 ± 0.073	1.38 ± 0.020	0.109	Tyr 78
		+16	769.82 (2+)	0.10 ± 0.012	0.16 ± 0.006	0.002	Tyr 78
**ACRVNHVTL (79-87)**	535.28 (2+) 357.19 (3+)	+16	362.52 (3+)	0.16 ± 0.005	0.17 ± 0.003	0.041	His 84
**SQPKIVKW (88-95)**	493.29 (2+)	+16	501.29 (2+)	0.49 ± 0.055	0.43 ± 0.014	0.141	Unassigned
		+16	501.29 (2+)	0.44 ± 0.058	0.34 ± 0.004	0.041	Unassigned
		+16	501.29 (2+)	0.19 ± 0.034	0.22 ± 0.010	0.217	Lys 95
		+16	501.29 (2+)	0.28 ± 0.086	0.40 ± 0.155	0.306	Trp 95
		+16	501.29 (2+)	0.32 ± 0.128	0.53 ± 0.126	0.113	Trp 95
		+16	501.29 (2+)	0.35 ± 0.116	0.40 ± 0.014	0.499	Trp 95
		+16	501.29 (2+)	0.09 ± 0.009	0.20 ± 0.092	0.108	Trp 95

Table S3. Raw HDX data for wild-type and D76N β_2m. State data csv file exported from DynamX v3.0.

Start	End	Sequence	MaxUptake	MHP	State	Exposure	Center	Center SD	Uptake	Uptake SD	RT	RT SD
1	10	MIQRTPKIQV	8	1213.709	Wild-type	0	1214.699	0.110156	0	0	5.229243	0.007765
1	10	MIQRTPKIQV	8	1213.709	Wild-type	0.5	1216.927	0.052013	2.22757	0.121818	5.216501	0.003139
1	10	MIQRTPKIQV	8	1213.709	Wild-type	1	1217.047	0.020263	2.348262	0.112005	5.218931	0.009556
1	10	MIQRTPKIQV	8	1213.709	Wild-type	2	1217.073	0.031444	2.37354	0.114556	5.217509	0.006497
1	10	MIQRTPKIQV	8	1213.709	Wild-type	30	1217.312	0.094569	2.612755	0.145182	5.227833	0.005937
1	10	MIQRTPKIQV	8	1213.709	Wild-type	120	1217.689	0.041917	2.989634	0.117862	5.220066	0.006368
1	23	MIQRTPKIQVYSRHPAENGKSNF	20	2701.399	Wild-type	0	2703.178	0.173653	0	0	4.742606	0.013523
1	23	MIQRTPKIQVYSRHPAENGKSNF	20	2701.399	Wild-type	0.5	2707.268	0.089718	4.089328	0.19546	4.720508	0.015203
1	23	MIQRTPKIQVYSRHPAENGKSNF	20	2701.399	Wild-type	1	2707.831	0.153422	4.652811	0.231719	4.730679	0.011391
1	23	MIQRTPKIQVYSRHPAENGKSNF	20	2701.399	Wild-type	2	2708.129	0.114033	4.950551	0.207747	4.737326	0.011429
1	23	MIQRTPKIQVYSRHPAENGKSNF	20	2701.399	Wild-type	30	2708.912	0.157972	5.733975	0.234756	4.740429	0.014761
1	23	MIQRTPKIQVYSRHPAENGKSNF	20	2701.399	Wild-type	120	2709.492	0.10558	6.314057	0.20323	4.730613	0.020098
2	10	IQRTPKIQV	7	1082.668	Wild-type	0	1083.255	0.048997	0	0	5.224111	0.009737
2	10	IQRTPKIQV	7	1082.668	Wild-type	0.5	1085.467	0.032135	2.211921	0.058595	5.216922	0.009231
2	10	IQRTPKIQV	7	1082.668	Wild-type	1	1085.57	0.049659	2.314294	0.069761	5.222102	0.009921
2	10	IQRTPKIQV	7	1082.668	Wild-type	2	1085.579	0.040763	2.323559	0.063736	5.218181	0.011893
2	10	IQRTPKIQV	7	1082.668	Wild-type	30	1085.806	0.05891	2.550689	0.076623	5.230967	0.008805
2	10	IQRTPKIQV	7	1082.668	Wild-type	120	1086.178	0.070308	2.922814	0.085697	5.217113	0.002029
4	10	RTPKIQV	5	841.5254	Wild-type	0	841.9375	0.025006	0	0	5.239659	0.002771
4	10	RTPKIQV	5	841.5254	Wild-type	0.5	843.7894	0.016773	1.851816	0.03011	5.22825	0.002531
4	10	RTPKIQV	5	841.5254	Wild-type	1	843.805	0.033239	1.867478	0.041595	5.231006	0.002534
4	10	RTPKIQV	5	841.5254	Wild-type	2	843.8558	0.022283	1.918272	0.033494	5.226953	0.003116
4	10	RTPKIQV	5	841.5254	Wild-type	30	843.992	0.024052	2.054457	0.034696	5.237227	0.003951
4	10	RTPKIQV	5	841.5254	Wild-type	120	844.2848	0.017523	2.347254	0.030535	5.233119	0.003387
6	10	PKIQV	3	584.3766	Wild-type	0	584.7122	0.010537	0	0	5.237819	0.001969
6	10	PKIQV	3	584.3766	Wild-type	0.5	585.6958	0.010443	0.983587	0.014835	5.227337	0.003955
6	10	PKIQV	3	584.3766	Wild-type	1	585.706	0.013134	0.993812	0.016838	5.225572	0.002075

6	10	PKIQV	3	584.3766	Wild-type	2	585.7397	0.022927	1.027521	0.025233	5.226057	0.005902
6	10	PKIQV	3	584.3766	Wild-type	30	585.8218	0.016283	1.109606	0.019395	5.232328	0.004502
6	10	PKIQV	3	584.3766	Wild-type	120	586.0565	0.027386	1.344333	0.029344	5.236265	0.00362
11	23	YSRHPAENGKSNF	11	1506.708	Wild-type	0	1507.564	0.045963	0	0	4.030765	0.023494
11	23	YSRHPAENGKSNF	11	1506.708	Wild-type	0.5	1509.838	0.051204	2.273441	0.068807	4.005224	0.010268
11	23	YSRHPAENGKSNF	11	1506.708	Wild-type	1	1510.251	0.037177	2.686715	0.059116	4.0022	0.013856
11	23	YSRHPAENGKSNF	11	1506.708	Wild-type	2	1510.513	0.040679	2.948877	0.061379	3.998101	0.006543
11	23	YSRHPAENGKSNF	11	1506.708	Wild-type	30	1510.723	0.092119	3.158665	0.102949	4.005757	0.021079
11	23	YSRHPAENGKSNF	11	1506.708	Wild-type	120	1510.938	0.08571	3.37417	0.097257	3.997953	0.01836
11	26	YSRHPAENGKSNFLNC	14	1836.845	Wild-type	0	1838.01	0.17075	0	0	5.332149	0.006823
11	26	YSRHPAENGKSNFLNC	14	1836.845	Wild-type	0.5	1839.944	0.059739	1.933691	0.180898	5.320745	0.005838
11	26	YSRHPAENGKSNFLNC	14	1836.845	Wild-type	1	1840.303	0.064195	2.292508	0.182418	5.321015	0.006705
11	26	YSRHPAENGKSNFLNC	14	1836.845	Wild-type	2	1840.489	0.241189	2.478688	0.295513	5.319301	0.00713
11	26	YSRHPAENGKSNFLNC	14	1836.845	Wild-type	30	1840.788	0.09294	2.778304	0.194405	5.325065	0.00624
11	26	YSRHPAENGKSNFLNC	14	1836.845	Wild-type	120	1840.93	0.106282	2.920338	0.201125	5.324339	0.006258
27	35	YVSGFHPSD	7	1008.442	Wild-type	0	1008.996	0.02332	0	0	5.151677	0.006176
27	35	YVSGFHPSD	7	1008.442	Wild-type	0.5	1009.762	0.029629	0.766376	0.037705	5.141742	0.005624
27	35	YVSGFHPSD	7	1008.442	Wild-type	1	1009.791	0.05686	0.795168	0.061456	5.138037	0.00583
27	35	YVSGFHPSD	7	1008.442	Wild-type	2	1009.866	0.053258	0.870033	0.058139	5.135919	0.009433
27	35	YVSGFHPSD	7	1008.442	Wild-type	30	1010.21	0.106938	1.213974	0.109451	5.14422	0.006995
27	35	YVSGFHPSD	7	1008.442	Wild-type	120	1010.498	0.057986	1.502709	0.062499	5.147817	0.008109
27	37	YVSGFHPSDIE	9	1250.569	Wild-type	0	1251.283	0.032999	0	0	5.882547	0.005044
27	37	YVSGFHPSDIE	9	1250.569	Wild-type	0.5	1251.772	0.020551	0.489286	0.038875	5.87549	0.006826
27	37	YVSGFHPSDIE	9	1250.569	Wild-type	1	1251.844	0.021328	0.560529	0.039291	5.880749	0.010427
27	37	YVSGFHPSDIE	9	1250.569	Wild-type	2	1251.848	0.017899	0.564952	0.037541	5.876592	0.008331
27	37	YVSGFHPSDIE	9	1250.569	Wild-type	30	1252.162	0.038379	0.878874	0.050615	5.885993	0.006033
27	37	YVSGFHPSDIE	9	1250.569	Wild-type	120	1252.584	0.084602	1.300572	0.09081	5.879303	0.008135
27	38	YVSGFHPSDIEV	10	1349.637	Wild-type	0	1350.05	0.011331	0	0	6.499181	0.002418
27	38	YVSGFHPSDIEV	10	1349.637	Wild-type	0.5	1350.759	0.033537	0.709543	0.035399	6.49309	0.003374
27	38	YVSGFHPSDIEV	10	1349.637	Wild-type	1	1350.811	0.061855	0.761645	0.062884	6.496167	0.003705

27	38	YVSGFHPSDIEV	10	1349.637	Wild-type	2	1350.832	0.019027	0.782511	0.022145	6.494154	0.002391
27	38	YVSGFHPSDIEV	10	1349.637	Wild-type	30	1351.338	0.066844	1.287955	0.067797	6.497715	0.004144
27	38	YVSGFHPSDIEV	10	1349.637	Wild-type	120	1351.877	0.133819	1.826896	0.134298	6.497496	0.003026
29	35	SGFHPSD	5	746.3104	Wild-type	0	746.7063	0.018851	0	0	5.170001	0.006592
29	35	SGFHPSD	5	746.3104	Wild-type	0.5	747.3518	0.009964	0.645493	0.021323	5.161929	0.004642
29	35	SGFHPSD	5	746.3104	Wild-type	1	747.4043	0.014649	0.698005	0.023874	5.161931	0.002338
29	35	SGFHPSD	5	746.3104	Wild-type	2	747.4088	0.032692	0.702451	0.037738	5.161923	0.003463
29	35	SGFHPSD	5	746.3104	Wild-type	30	747.6654	0.051436	0.959055	0.054782	5.169271	0.005167
29	35	SGFHPSD	5	746.3104	Wild-type	120	747.8481	0.034643	1.141731	0.03944	5.163618	0.002726
36	39	IEVD	3	475.2399	Wild-type	0	475.4326	0.036761	0	0	4.695695	0.006396
36	39	IEVD	3	475.2399	Wild-type	0.5	475.4398	0.039157	0.007142	0.053709	4.685644	0.005259
36	39	IEVD	3	475.2399	Wild-type	1	475.4888	0.048342	0.056198	0.060731	4.695862	0.006376
36	39	IEVD	3	475.2399	Wild-type	2	475.5067	0.06306	0.074016	0.072993	4.688796	0.00449
36	39	IEVD	3	475.2399	Wild-type	30	475.7218	0.075033	0.289129	0.083554	4.694839	0.002526
36	39	IEVD	3	475.2399	Wild-type	120	475.9415	0.029335	0.508818	0.047031	4.700346	0.007816
36	40	IEVDL	4	588.3239	Wild-type	0	588.6093	0.010492	0	0	6.681435	0.006506
36	40	IEVDL	4	588.3239	Wild-type	0.5	588.8049	0.010707	0.195571	0.014991	6.673932	0.007843
36	40	IEVDL	4	588.3239	Wild-type	1	588.8139	0.020559	0.204521	0.023082	6.682516	0.003824
36	40	IEVDL	4	588.3239	Wild-type	2	588.8249	0.017925	0.215585	0.02077	6.67284	0.006403
36	40	IEVDL	4	588.3239	Wild-type	30	588.9286	0.021037	0.319252	0.023509	6.680051	0.005781
36	40	IEVDL	4	588.3239	Wild-type	120	589.1721	0.024368	0.562722	0.026531	6.670657	0.006939
36	41	IEVDLL	5	701.408	Wild-type	0	701.8555	0.022902	0	0	7.567922	0.002016
36	41	IEVDLL	5	701.408	Wild-type	0.5	701.9884	0.03959	0.132897	0.045737	7.558953	0.003892
36	41	IEVDLL	5	701.408	Wild-type	1	702.025	0.027481	0.169448	0.035773	7.557954	0.00124
36	41	IEVDLL	5	701.408	Wild-type	2	702.0252	0.029375	0.169699	0.037247	7.558298	0.003905
36	41	IEVDLL	5	701.408	Wild-type	30	702.1118	0.017804	0.256288	0.029008	7.565747	0.005082
36	41	IEVDLL	5	701.408	Wild-type	120	702.3584	0.058208	0.502898	0.062551	7.562408	0.003386
36	56	IEVDLLKNGERIEKVEHSDLS	20	2423.278	Wild-type	0	2424.733	0.129963	0	0	5.924269	0.01181
36	56	IEVDLLKNGERIEKVEHSDLS	20	2423.278	Wild-type	0.5	2428.508	0.0936	3.774518	0.160161	5.90267	0.006549
36	56	IEVDLLKNGERIEKVEHSDLS	20	2423.278	Wild-type	1	2429.094	0.116335	4.360337	0.174425	5.899197	0.005292

36	56	IEVDLLKNGERIEKVEHSDLS	20	2423.278	Wild-type	2	2429.413	0.090427	4.680065	0.158327	5.898199	0.005782
36	56	IEVDLLKNGERIEKVEHSDLS	20	2423.278	Wild-type	30	2430.298	0.172694	5.564976	0.216133	5.90442	0.006758
36	56	IEVDLLKNGERIEKVEHSDLS	20	2423.278	Wild-type	120	2430.735	0.097565	6.001487	0.16251	5.899116	0.007857
36	57	IEVDLLKNGERIEKVEHSDLSF	21	2570.346	Wild-type	0	2571.901	0.087798	0	0	6.500686	0.005672
36	57	IEVDLLKNGERIEKVEHSDLSF	21	2570.346	Wild-type	0.5	2575.719	0.109088	3.818396	0.14003	6.480742	0.00989
36	57	IEVDLLKNGERIEKVEHSDLSF	21	2570.346	Wild-type	1	2576.42	0.132982	4.518856	0.159351	6.478239	0.005917
36	57	IEVDLLKNGERIEKVEHSDLSF	21	2570.346	Wild-type	2	2576.782	0.107743	4.881435	0.138985	6.478364	0.010199
36	57	IEVDLLKNGERIEKVEHSDLSF	21	2570.346	Wild-type	30	2577.705	0.1504	5.803501	0.174151	6.487378	0.0069
36	57	IEVDLLKNGERIEKVEHSDLSF	21	2570.346	Wild-type	120	2578.047	0.139532	6.146236	0.164856	6.490951	0.003406
38	55	VDLLKNGERIEKVEHSDL	17	2094.119	Wild-type	0	2095.469	0.16982	0	0	5.658236	0.007425
38	55	VDLLKNGERIEKVEHSDL	17	2094.119	Wild-type	0.5	2098.946	0.131761	3.476904	0.214941	5.634796	0.007728
38	55	VDLLKNGERIEKVEHSDL	17	2094.119	Wild-type	1	2099.483	0.140079	4.01381	0.220139	5.631019	0.008005
38	55	VDLLKNGERIEKVEHSDL	17	2094.119	Wild-type	2	2099.727	0.084943	4.257237	0.189879	5.626704	0.009114
38	55	VDLLKNGERIEKVEHSDL	17	2094.119	Wild-type	30	2100.082	0.082957	4.612453	0.188999	5.639348	0.009639
38	55	VDLLKNGERIEKVEHSDL	17	2094.119	Wild-type	120	2100.328	0.063414	4.858941	0.181273	5.646067	0.005497
38	56	VDLLKNGERIEKVEHSDLS	18	2181.151	Wild-type	0	2182.431	0.126688	0	0	5.510468	0.00866
38	56	VDLLKNGERIEKVEHSDLS	18	2181.151	Wild-type	0.5	2186.232	0.093327	3.800895	0.157353	5.486638	0.005524
38	56	VDLLKNGERIEKVEHSDLS	18	2181.151	Wild-type	1	2186.82	0.071925	4.389391	0.145681	5.485957	0.006093
38	56	VDLLKNGERIEKVEHSDLS	18	2181.151	Wild-type	2	2187.258	0.131156	4.827361	0.182351	5.486536	0.011054
38	56	VDLLKNGERIEKVEHSDLS	18	2181.151	Wild-type	30	2187.855	0.124166	5.42478	0.17739	5.491611	0.008124
38	56	VDLLKNGERIEKVEHSDLS	18	2181.151	Wild-type	120	2188.179	0.083931	5.748096	0.151968	5.488414	0.010382
39	57	DLLKNGERIEKVEHSDLSF	18	2229.151	Wild-type	0	2230.364	0.043567	0	0	6.150778	0.013492
39	57	DLLKNGERIEKVEHSDLSF	18	2229.151	Wild-type	0.5	2234.252	0.053604	3.88785	0.069076	6.12161	0.007673
39	57	DLLKNGERIEKVEHSDLSF	18	2229.151	Wild-type	1	2234.95	0.101832	4.585802	0.11076	6.126575	0.005843
39	57	DLLKNGERIEKVEHSDLSF	18	2229.151	Wild-type	2	2235.383	0.076416	5.019458	0.087963	6.119119	0.004068
39	57	DLLKNGERIEKVEHSDLSF	18	2229.151	Wild-type	30	2236.259	0.150473	5.895455	0.156653	6.132307	0.007957
39	57	DLLKNGERIEKVEHSDLSF	18	2229.151	Wild-type	120	2236.479	0.077836	6.114818	0.0892	6.136281	0.01196
40	55	LLKNGERIEKVEHSDL	15	1880.024	Wild-type	0	1881.062	0.114025	0	0	4.993127	0.047343
40	55	LLKNGERIEKVEHSDL	15	1880.024	Wild-type	0.5	1884.083	0.075553	3.020762	0.136784	4.947628	0.015687
40	55	LLKNGERIEKVEHSDL	15	1880.024	Wild-type	1	1884.54	0.071192	3.47751	0.134425	4.948257	0.02124

40	55	LLKNGERIEKVEHSDL	15	1880.024	Wild-type	2	1884.781	0.092567	3.719148	0.146868	4.935539	0.015047
40	55	LLKNGERIEKVEHSDL	15	1880.024	Wild-type	30	1884.995	0.204817	3.932758	0.234418	4.952678	0.025315
40	55	LLKNGERIEKVEHSDL	15	1880.024	Wild-type	120	1885.278	0.141365	4.216157	0.18162	4.972665	0.015789
40	56	LLKNGERIEKVEHSDLS	16	1967.056	Wild-type	0	1968.055	0.072889	0	0	4.723692	0.021099
40	56	LLKNGERIEKVEHSDLS	16	1967.056	Wild-type	0.5	1971.474	0.085525	3.418849	0.112372	4.674503	0.00654
40	56	LLKNGERIEKVEHSDLS	16	1967.056	Wild-type	1	1971.919	0.137788	3.863381	0.155879	4.681907	0.009333
40	56	LLKNGERIEKVEHSDLS	16	1967.056	Wild-type	2	1972.241	0.135276	4.185906	0.153663	4.672975	0.010167
40	56	LLKNGERIEKVEHSDLS	16	1967.056	Wild-type	30	1972.894	0.074402	4.838948	0.104157	4.69527	0.011928
40	56	LLKNGERIEKVEHSDLS	16	1967.056	Wild-type	120	1973.093	0.062612	5.038109	0.096089	4.699976	0.008009
40	57	LLKNGERIEKVEHSDLSF	17	2114.124	Wild-type	0	2115.13	0.069353	0	0	5.644397	0.010804
40	57	LLKNGERIEKVEHSDLSF	17	2114.124	Wild-type	0.5	2118.712	0.100258	3.581173	0.121908	5.612885	0.009693
40	57	LLKNGERIEKVEHSDLSF	17	2114.124	Wild-type	1	2119.373	0.042781	4.242146	0.081487	5.623402	0.023674
40	57	LLKNGERIEKVEHSDLSF	17	2114.124	Wild-type	2	2119.737	0.05629	4.606454	0.089322	5.602166	0.008792
40	57	LLKNGERIEKVEHSDLSF	17	2114.124	Wild-type	30	2120.547	0.066751	5.41696	0.096258	5.623803	0.007438
40	57	LLKNGERIEKVEHSDLSF	17	2114.124	Wild-type	120	2120.82	0.06936	5.689635	0.098085	5.623783	0.012269
41	55	LKNGERIEKVEHSDL	14	1766.94	Wild-type	0	1768.007	0.07225	0	0	4.628079	0.006815
41	55	LKNGERIEKVEHSDL	14	1766.94	Wild-type	0.5	1770.811	0.060472	2.804337	0.094218	4.594922	0.008602
41	55	LKNGERIEKVEHSDL	14	1766.94	Wild-type	1	1771.228	0.052924	3.221134	0.089561	4.58532	0.007314
41	55	LKNGERIEKVEHSDL	14	1766.94	Wild-type	2	1771.428	0.080247	3.421264	0.10798	4.589089	0.00855
41	55	LKNGERIEKVEHSDL	14	1766.94	Wild-type	30	1771.769	0.069886	3.762074	0.10052	4.600366	0.008503
41	55	LKNGERIEKVEHSDL	14	1766.94	Wild-type	120	1771.833	0.051232	3.825975	0.088571	4.606667	0.007911
41	56	LKNGERIEKVEHSDLS	15	1853.972	Wild-type	0	1854.995	0.048789	0	0	4.429675	0.013891
41	56	LKNGERIEKVEHSDLS	15	1853.972	Wild-type	0.5	1858.091	0.065797	3.096183	0.081912	4.384598	0.010578
41	56	LKNGERIEKVEHSDLS	15	1853.972	Wild-type	1	1858.477	0.054178	3.482071	0.072908	4.396365	0.014037
41	56	LKNGERIEKVEHSDLS	15	1853.972	Wild-type	2	1858.815	0.049836	3.820891	0.069742	4.382133	0.010923
41	56	LKNGERIEKVEHSDLS	15	1853.972	Wild-type	30	1859.403	0.088125	4.408903	0.100729	4.399956	0.014478
41	56	LKNGERIEKVEHSDLS	15	1853.972	Wild-type	120	1859.557	0.134515	4.56239	0.14309	4.395497	0.015967
41	57	LKNGERIEKVEHSDLSF	16	2001.04	Wild-type	0	2002.318	0.125649	0	0	5.469909	0.013117
41	57	LKNGERIEKVEHSDLSF	16	2001.04	Wild-type	0.5	2005.57	0.104351	3.252128	0.163331	5.432427	0.015499
41	57	LKNGERIEKVEHSDLSF	16	2001.04	Wild-type	1	2006.077	0.040895	3.758541	0.132137	5.417272	0.012282

41	57	LKNGERIEKVEHSDLSF	16	2001.04	Wild-type	2	2006.456	0.089321	4.138032	0.154162	5.431317	0.018629
41	57	LKNGERIEKVEHSDLSF	16	2001.04	Wild-type	30	2007.125	0.09545	4.806986	0.157792	5.442561	0.017779
41	57	LKNGERIEKVEHSDLSF	16	2001.04	Wild-type	120	2007.473	0.123172	5.154857	0.175952	5.473092	0.018735
56	63	SFSKDWSF	7	1003.452	Wild-type	0	1004.149	0.051097	0	0	7.170587	0.007393
56	63	SFSKDWSF	7	1003.452	Wild-type	0.5	1005.498	0.029149	1.348462	0.058827	7.165932	0.009591
56	63	SFSKDWSF	7	1003.452	Wild-type	1	1005.749	0.067091	1.59961	0.084334	7.166143	0.003401
56	63	SFSKDWSF	7	1003.452	Wild-type	2	1005.946	0.055233	1.796637	0.075244	7.156014	0.004592
56	63	SFSKDWSF	7	1003.452	Wild-type	30	1006.513	0.07948	2.363868	0.094488	7.16813	0.011707
56	63	SFSKDWSF	7	1003.452	Wild-type	120	1006.877	0.048646	2.727172	0.07055	7.166091	0.003674
57	61	FSKDW	4	682.3195	Wild-type	0	682.763	0.046295	0	0	6.805427	0.181855
57	61	FSKDW	4	682.3195	Wild-type	0.5	683.3136	0.029132	0.550604	0.054699	6.688492	0.004449
57	61	FSKDW	4	682.3195	Wild-type	1	683.4517	0.008231	0.688741	0.047021	6.690259	0.003229
57	61	FSKDW	4	682.3195	Wild-type	2	683.5739	0.00965	0.810937	0.04729	6.686934	0.005185
57	61	FSKDW	4	682.3195	Wild-type	30	683.9661	0.007356	1.203136	0.046876	6.691316	0.001508
57	61	FSKDW	4	682.3195	Wild-type	120	684.2116	0.017286	1.448579	0.049417	6.686803	0.003805
57	63	FSKDWSF	6	916.4199	Wild-type	0	917.0626	0.033038	0	0	7.059535	0.005654
57	63	FSKDWSF	6	916.4199	Wild-type	0.5	918.116	0.023289	1.053429	0.040421	7.051	0.006392
57	63	FSKDWSF	6	916.4199	Wild-type	1	918.3064	0.013864	1.24382	0.035829	7.045759	0.007623
57	63	FSKDWSF	6	916.4199	Wild-type	2	918.476	0.010595	1.413361	0.034695	7.049784	0.008941
57	63	FSKDWSF	6	916.4199	Wild-type	30	918.9546	0.027676	1.891972	0.043098	7.058093	0.007878
57	63	FSKDWSF	6	916.4199	Wild-type	120	919.2716	0.032622	2.208983	0.046429	7.05596	0.008105
58	62	SKDWS	4	622.2831	Wild-type	0	622.5111	0.005747	0	0	6.693337	0.003573
58	62	SKDWS	4	622.2831	Wild-type	0.5	622.9813	0.020949	0.470259	0.021723	6.688709	0.004548
58	62	SKDWS	4	622.2831	Wild-type	1	623.1156	0.016861	0.604571	0.017814	6.690497	0.002374
58	62	SKDWS	4	622.2831	Wild-type	2	623.2193	0.029234	0.708199	0.029793	6.686732	0.004101
58	62	SKDWS	4	622.2831	Wild-type	30	623.5472	0.026089	1.036176	0.026715	6.691753	0.003099
58	62	SKDWS	4	622.2831	Wild-type	120	623.786	0.021328	1.274894	0.022089	6.687423	0.004696
58	63	SKDWSF	5	769.3515	Wild-type	0	769.804	0.028892	0	0	6.686241	0.00313
58	63	SKDWSF	5	769.3515	Wild-type	0.5	770.4808	0.024184	0.676767	0.037678	6.677662	0.006898
58	63	SKDWSF	5	769.3515	Wild-type	1	770.6449	0.034072	0.840895	0.044673	6.671721	0.004611

58	63	SKDWSF	5	769.3515	Wild-type	2	770.818	0.037739	1.013982	0.047528	6.675325	0.005206
58	63	SKDWSF	5	769.3515	Wild-type	30	771.3104	0.024779	1.506365	0.038063	6.684063	0.009503
58	63	SKDWSF	5	769.3515	Wild-type	120	771.6329	0.049404	1.828939	0.057232	6.685461	0.00553
59	63	KDWSF	4	682.3195	Wild-type	0	682.7	0.023501	0	0	6.903986	0.198585
59	63	KDWSF	4	682.3195	Wild-type	0.5	683.5707	0.020906	0.870699	0.031454	7.081375	0.008812
59	63	KDWSF	4	682.3195	Wild-type	1	683.7178	0.020423	1.017741	0.031135	7.079706	0.001939
59	63	KDWSF	4	682.3195	Wild-type	2	683.848	0.013969	1.147959	0.027339	7.080964	0.005673
59	63	KDWSF	4	682.3195	Wild-type	30	684.2087	0.03511	1.508645	0.042249	7.08699	0.009826
59	63	KDWSF	4	682.3195	Wild-type	120	684.4578	0.037213	1.757803	0.044012	7.090877	0.003307
60	63	DWSF	3	554.2245	Wild-type	0	554.5049	0.005004	0	0	6.694761	0.002597
60	63	DWSF	3	554.2245	Wild-type	0.5	554.9788	0.022659	0.473931	0.023205	6.689023	0.002401
60	63	DWSF	3	554.2245	Wild-type	1	555.118	0.014871	0.61309	0.01569	6.689998	0.001658
60	63	DWSF	3	554.2245	Wild-type	2	555.2192	0.024014	0.714293	0.02453	6.687558	0.002979
60	63	DWSF	3	554.2245	Wild-type	30	555.5767	0.045415	1.071796	0.045689	6.68983	0.002968
60	63	DWSF	3	554.2245	Wild-type	120	555.7822	0.02941	1.277302	0.029833	6.689533	0.003945
63	67	FYLLY	4	718.381	Wild-type	0	718.7543	0.015679	0	0	8.487907	0.00663
63	67	FYLLY	4	718.381	Wild-type	0.5	718.7661	0.01421	0.011853	0.02116	8.47931	0.003991
63	67	FYLLY	4	718.381	Wild-type	1	718.7962	0.015387	0.041988	0.021968	8.478259	0.007179
63	67	FYLLY	4	718.381	Wild-type	2	718.777	0.015428	0.022723	0.021996	8.471966	0.005792
63	67	FYLLY	4	718.381	Wild-type	30	718.7795	0.011953	0.025222	0.019716	8.479384	0.003599
63	67	FYLLY	4	718.381	Wild-type	120	718.7915	0.012017	0.037255	0.019754	8.475742	0.004919
64	67	YLLY	3	571.3126	Wild-type	0	571.6984	0.013675	0	0	7.428169	0.003963
64	67	YLLY	3	571.3126	Wild-type	0.5	571.7555	0.020134	0.057157	0.024339	7.418172	0.006519
64	67	YLLY	3	571.3126	Wild-type	1	571.7594	0.013315	0.061055	0.019087	7.419137	0.001746
64	67	YLLY	3	571.3126	Wild-type	2	571.7405	0.005514	0.042058	0.014745	7.415618	0.00563
64	67	YLLY	3	571.3126	Wild-type	30	571.7217	0.024421	0.023336	0.027989	7.419908	0.00657
64	67	YLLY	3	571.3126	Wild-type	120	571.7197	0.024358	0.021338	0.027934	7.417576	0.007555
64	68	YLLYY	4	734.376	Wild-type	0	734.7517	0.040057	0	0	7.879937	0.003223
64	68	YLLYY	4	734.376	Wild-type	0.5	734.7547	0.043073	0.003025	0.05882	7.862638	0.002549
64	68	YLLYY	4	734.376	Wild-type	1	734.7739	0.034193	0.022222	0.052667	7.859097	0.004127

64	68	YLLYY	4	734.376	Wild-type	2	734.7659	0.018833	0.014195	0.044264	7.858931	0.004259
64	68	YLLYY	4	734.376	Wild-type	30	734.796	0.032747	0.044299	0.051739	7.861516	0.003332
64	68	YLLYY	4	734.376	Wild-type	120	734.8191	0.067623	0.067428	0.078596	7.858501	0.008271
65	68	LLYY	3	571.3126	Wild-type	0	571.6055	0.008227	0	0	7.056081	0.002468
65	68	LLYY	3	571.3126	Wild-type	0.5	571.6415	0.009618	0.035996	0.012657	7.047083	0.004395
65	68	LLYY	3	571.3126	Wild-type	1	571.644	0.005251	0.038482	0.00976	7.047602	0.003017
65	68	LLYY	3	571.3126	Wild-type	2	571.6426	0.016569	0.037103	0.018499	7.040026	0.001424
65	68	LLYY	3	571.3126	Wild-type	30	571.63	0.013992	0.024529	0.016231	7.046752	0.003853
65	68	LLYY	3	571.3126	Wild-type	120	571.6287	0.015374	0.023235	0.017437	7.042436	0.006116
66	71	LYYTEF	5	835.3872	Wild-type	0	835.8102	0.023969	0	0	7.479241	0.005094
66	71	LYYTEF	5	835.3872	Wild-type	0.5	835.9964	0.02571	0.186163	0.03515	7.473088	0.002038
66	71	LYYTEF	5	835.3872	Wild-type	1	836.0405	0.035163	0.230302	0.042556	7.470708	0.001855
66	71	LYYTEF	5	835.3872	Wild-type	2	836.1132	0.024663	0.302944	0.034392	7.465755	0.004237
66	71	LYYTEF	5	835.3872	Wild-type	30	836.4489	0.029009	0.638695	0.037631	7.47242	0.003549
66	71	LYYTEF	5	835.3872	Wild-type	120	836.5037	0.026992	0.693464	0.036099	7.469194	0.004177
67	70	YYTE	3	575.2348	Wild-type	0	575.5637	0.014674	0	0	4.717291	0.002237
67	70	YYTE	3	575.2348	Wild-type	0.5	575.7256	0.017477	0.161826	0.02282	4.710954	0.002678
67	70	YYTE	3	575.2348	Wild-type	1	575.777	0.020424	0.2133	0.025149	4.71176	0.003302
67	70	YYTE	3	575.2348	Wild-type	2	575.8477	0.004671	0.283949	0.0154	4.70884	0.003394
67	70	YYTE	3	575.2348	Wild-type	30	576.139	0.005165	0.575265	0.015557	4.712401	0.004125
67	70	YYTE	3	575.2348	Wild-type	120	576.1857	0.013597	0.621985	0.020005	4.714993	0.001527
67	71	YYTEF	4	722.3032	Wild-type	0	722.7689	0.025124	0	0	6.94309	0.002802
67	71	YYTEF	4	722.3032	Wild-type	0.5	722.966	0.005314	0.197098	0.02568	6.934291	0.003843
67	71	YYTEF	4	722.3032	Wild-type	1	723.0087	0.015499	0.239807	0.02952	6.934302	0.001555
67	71	YYTEF	4	722.3032	Wild-type	2	723.0614	0.015233	0.292515	0.029381	6.928956	0.002995
67	71	YYTEF	4	722.3032	Wild-type	30	723.3205	0.018545	0.55159	0.031227	6.935049	0.005438
67	71	YYTEF	4	722.3032	Wild-type	120	723.3419	0.013423	0.573073	0.028485	6.932349	0.0037
67	79	YYTEFTPTEKDEY	11	1685.722	Wild-type	0	1686.645	0.053346	0	0	6.193201	0.002825
67	79	YYTEFTPTEKDEY	11	1685.722	Wild-type	0.5	1688.465	0.05201	1.820396	0.074504	6.195477	0.007599
67	79	YYTEFTPTEKDEY	11	1685.722	Wild-type	1	1688.797	0.066874	2.152103	0.085545	6.193939	0.003497

67	79	YYTEFTPTEKDEY	11	1685.722	Wild-type	2	1688.947	0.059791	2.302476	0.08013	6.195345	0.006453
67	79	YYTEFTPTEKDEY	11	1685.722	Wild-type	30	1689.891	0.125131	3.246487	0.136028	6.193824	0.00727
67	79	YYTEFTPTEKDEY	11	1685.722	Wild-type	120	1690.553	0.078356	3.907894	0.094792	6.190218	0.003985
68	71	YTEF	3	559.2399	Wild-type	0	559.601	0.017192	0	0	6.318462	0.005083
68	71	YTEF	3	559.2399	Wild-type	0.5	559.7749	0.034129	0.173835	0.038215	6.312008	0.004402
68	71	YTEF	3	559.2399	Wild-type	1	559.8292	0.040839	0.228194	0.04431	6.315024	0.005542
68	71	YTEF	3	559.2399	Wild-type	2	559.8621	0.030337	0.261096	0.03487	6.309176	0.006035
68	71	YTEF	3	559.2399	Wild-type	30	560.1081	0.009767	0.507123	0.019773	6.31826	0.002909
68	71	YTEF	3	559.2399	Wild-type	120	560.1375	0.009629	0.536532	0.019705	6.314687	0.002823
68	78	YTEFTPTEKDE	9	1359.595	Wild-type	0	1360.301	0.068753	0	0	5.20683	0.002507
68	78	YTEFTPTEKDE	9	1359.595	Wild-type	0.5	1362.04	0.029449	1.739583	0.074794	5.197984	0.004165
68	78	YTEFTPTEKDE	9	1359.595	Wild-type	1	1362.394	0.065141	2.093784	0.094711	5.201588	0.007557
68	78	YTEFTPTEKDE	9	1359.595	Wild-type	2	1362.513	0.028108	2.212023	0.074277	5.200225	0.003809
68	78	YTEFTPTEKDE	9	1359.595	Wild-type	30	1363.207	0.032637	2.905956	0.076106	5.206383	0.00284
68	78	YTEFTPTEKDE	9	1359.595	Wild-type	120	1363.683	0.089316	3.382537	0.112713	5.202044	0.005306
68	80	YTEFTPTEKDEYA	11	1593.696	Wild-type	0	1594.665	0.037326	0	0	5.688583	0.007654
68	80	YTEFTPTEKDEYA	11	1593.696	Wild-type	0.5	1596.255	0.095996	1.590042	0.102998	5.691205	0.004412
68	80	YTEFTPTEKDEYA	11	1593.696	Wild-type	1	1596.551	0.069266	1.886386	0.078684	5.688142	0.00501
68	80	YTEFTPTEKDEYA	11	1593.696	Wild-type	2	1596.815	0.124929	2.149799	0.130386	5.690831	0.004349
68	80	YTEFTPTEKDEYA	11	1593.696	Wild-type	30	1597.633	0.171639	2.967816	0.175651	5.683803	0.004729
68	80	YTEFTPTEKDEYA	11	1593.696	Wild-type	120	1598.121	0.112405	3.456419	0.11844	5.68553	0.007878
68	81	YTEFTPTEKDEYAC	12	1696.705	Wild-type	0	1697.743	0.092062	0	0	5.91035	0.006689
68	81	YTEFTPTEKDEYAC	12	1696.705	Wild-type	0.5	1699.596	0.079405	1.852922	0.121575	5.911605	0.00471
68	81	YTEFTPTEKDEYAC	12	1696.705	Wild-type	1	1699.847	0.024718	2.104192	0.095323	5.905119	0.004245
68	81	YTEFTPTEKDEYAC	12	1696.705	Wild-type	2	1700.081	0.133788	2.338515	0.162402	5.90963	0.005575
68	81	YTEFTPTEKDEYAC	12	1696.705	Wild-type	30	1700.978	0.16822	3.234745	0.191763	5.908251	0.001709
68	81	YTEFTPTEKDEYAC	12	1696.705	Wild-type	120	1701.599	0.182873	3.85614	0.204738	5.906747	0.004746
69	78	TEFTPTEKDE	8	1196.532	Wild-type	0	1196.956	0.026811	0	0	4.777419	0.004663
69	78	TEFTPTEKDE	8	1196.532	Wild-type	0.5	1198.765	0.077207	1.80866	0.08173	4.780854	0.003727
69	78	TEFTPTEKDE	8	1196.532	Wild-type	1	1199.008	0.04375	2.0515	0.051312	4.782657	0.004475

69	78	TEFTPTEKDE	8	1196.532	Wild-type	2	1199.192	0.049578	2.236081	0.056364	4.780918	0.001787
69	78	TEFTPTEKDE	8	1196.532	Wild-type	30	1199.59	0.040469	2.633462	0.048545	4.78089	0.005851
69	78	TEFTPTEKDE	8	1196.532	Wild-type	120	1200.163	0.063622	3.206651	0.06904	4.77649	0.00445
69	80	TEFTPTEKDEYA	10	1430.632	Wild-type	0	1431.403	0.03494	0	0	5.343879	0.004054
69	80	TEFTPTEKDEYA	10	1430.632	Wild-type	0.5	1433.011	0.014821	1.607744	0.037953	5.335946	0.002747
69	80	TEFTPTEKDEYA	10	1430.632	Wild-type	1	1433.264	0.03416	1.860545	0.048864	5.33624	0.002282
69	80	TEFTPTEKDEYA	10	1430.632	Wild-type	2	1433.377	0.043135	1.973747	0.055511	5.337199	0.003111
69	80	TEFTPTEKDEYA	10	1430.632	Wild-type	30	1434.107	0.161722	2.704124	0.165453	5.340199	0.005841
69	80	TEFTPTEKDEYA	10	1430.632	Wild-type	120	1434.749	0.102678	3.345726	0.108459	5.343877	0.003796
69	81	TEFTPTEKDEYAC	11	1533.641	Wild-type	0	1534.444	0.052286	0	0	5.59318	0.004293
69	81	TEFTPTEKDEYAC	11	1533.641	Wild-type	0.5	1536.161	0.064696	1.717487	0.083183	5.584678	0.012946
69	81	TEFTPTEKDEYAC	11	1533.641	Wild-type	1	1536.425	0.027257	1.981677	0.058964	5.58253	0.008818
69	81	TEFTPTEKDEYAC	11	1533.641	Wild-type	2	1536.595	0.078065	2.151278	0.093957	5.582474	0.008735
69	81	TEFTPTEKDEYAC	11	1533.641	Wild-type	30	1537.322	0.215426	2.877972	0.22168	5.59066	0.003736
69	81	TEFTPTEKDEYAC	11	1533.641	Wild-type	120	1537.956	0.035963	3.512728	0.06346	5.587977	0.004135
82	100	RVNHVTLSQPKIVKWDRDM	17	2322.25	Wild-type	0	2323.599	0.07405	0	0	5.447825	0.009294
82	100	RVNHVTLSQPKIVKWDRDM	17	2322.25	Wild-type	0.5	2325.876	0.075042	2.276632	0.105426	5.427231	0.015586
82	100	RVNHVTLSQPKIVKWDRDM	17	2322.25	Wild-type	1	2326.314	0.07386	2.715542	0.104589	5.416331	0.00825
82	100	RVNHVTLSQPKIVKWDRDM	17	2322.25	Wild-type	2	2326.729	0.089479	3.129615	0.116146	5.422801	0.013366
82	100	RVNHVTLSQPKIVKWDRDM	17	2322.25	Wild-type	30	2327.847	0.138376	4.2482	0.156944	5.433211	0.008614
82	100	RVNHVTLSQPKIVKWDRDM	17	2322.25	Wild-type	120	2328.242	0.056565	4.643053	0.093183	5.421162	0.006152
91	100	PKIVKWDRDM	8	1287.688	Wild-type	0	1288.391	0.043896	0	0	5.401639	0.005738
91	100	PKIVKWDRDM	8	1287.688	Wild-type	0.5	1289.401	0.033011	1.00984	0.054923	5.386774	0.003573
91	100	PKIVKWDRDM	8	1287.688	Wild-type	1	1289.657	0.039464	1.266615	0.059027	5.392326	0.005092
91	100	PKIVKWDRDM	8	1287.688	Wild-type	2	1289.781	0.031976	1.390366	0.054308	5.386935	0.002723
91	100	PKIVKWDRDM	8	1287.688	Wild-type	30	1290.286	0.111428	1.895452	0.119762	5.397392	0.005123
91	100	PKIVKWDRDM	8	1287.688	Wild-type	120	1290.511	0.128439	2.120643	0.135733	5.396315	0.005389
94	100	VKWDRDM	6	949.456	Wild-type	0	950.0112	0.040563	0	0	5.376011	0.0028
94	100	VKWDRDM	6	949.456	Wild-type	0.5	950.8659	0.049045	0.854734	0.063646	5.36708	0.03733
94	100	VKWDRDM	6	949.456	Wild-type	1	951.1423	0.023951	1.131156	0.047106	5.366597	0.001128

94	100	VKWDRDM	6	949.456	Wild-type	2	951.2981	0.019138	1.286912	0.044851	5.364339	0.002433
94	100	VKWDRDM	6	949.456	Wild-type	30	951.5784	0.029272	1.567265	0.050022	5.37198	0.00181
94	100	VKWDRDM	6	949.456	Wild-type	120	951.697	0.031582	1.685812	0.051408	5.370827	0.004082
95	100	KWDRDM	5	850.3876	Wild-type	0	850.9531	0.038303	0	0	5.375086	0.005436
95	100	KWDRDM	5	850.3876	Wild-type	0.5	851.8063	0.050842	0.853211	0.063656	5.370724	0.002109
95	100	KWDRDM	5	850.3876	Wild-type	1	852.0435	0.066915	1.090391	0.077102	5.373317	0.004713
95	100	KWDRDM	5	850.3876	Wild-type	2	852.1459	0.061108	1.192752	0.07212	5.36747	0.003284
95	100	KWDRDM	5	850.3876	Wild-type	30	852.424	0.07772	1.470869	0.086646	5.377281	0.003021
95	100	KWDRDM	5	850.3876	Wild-type	120	852.6105	0.045008	1.657338	0.0591	5.376508	0.005425
1	10	MIQRTPKIQV	8	1213.709	D76N	0	1214.412	0.070198	0	0	5.231595	0.004103
1	10	MIQRTPKIQV	8	1213.709	D76N	0.5	1216.866	0.050103	2.454224	0.086244	5.227171	0.007118
1	10	MIQRTPKIQV	8	1213.709	D76N	1	1216.967	0.037774	2.555174	0.079716	5.225198	0.004359
1	10	MIQRTPKIQV	8	1213.709	D76N	2	1217.054	0.048119	2.64227	0.085106	5.232156	0.005228
1	10	MIQRTPKIQV	8	1213.709	D76N	30	1217.291	0.064831	2.879099	0.095555	5.238642	0.005248
1	10	MIQRTPKIQV	8	1213.709	D76N	120	1217.687	0.043998	3.27522	0.082846	5.243608	0.00357
1	23	MIQRTPKIQVYSRHPAENGKSNF	20	2701.399	D76N	0	2702.901	0.03874	0	0	4.739544	0.004972
1	23	MIQRTPKIQVYSRHPAENGKSNF	20	2701.399	D76N	0.5	2707.508	0.059545	4.607132	0.071038	4.728858	0.006495
1	23	MIQRTPKIQVYSRHPAENGKSNF	20	2701.399	D76N	1	2707.95	0.11885	5.049146	0.125005	4.730865	0.008989
1	23	MIQRTPKIQVYSRHPAENGKSNF	20	2701.399	D76N	2	2708.222	0.120967	5.321043	0.127019	4.737249	0.00962
1	23	MIQRTPKIQVYSRHPAENGKSNF	20	2701.399	D76N	30	2708.865	0.151137	5.964016	0.156023	4.738184	0.004882
1	23	MIQRTPKIQVYSRHPAENGKSNF	20	2701.399	D76N	120	2709.488	0.075151	6.587207	0.084548	4.742775	0.008462
2	10	IQRTPKIQV	7	1082.668	D76N	0	1083.213	0.009872	0	0	5.233356	0.00521
2	10	IQRTPKIQV	7	1082.668	D76N	0.5	1085.357	0.024463	2.144221	0.026379	5.229028	0.007571
2	10	IQRTPKIQV	7	1082.668	D76N	1	1085.478	0.026647	2.265108	0.028416	5.228946	0.008349
2	10	IQRTPKIQV	7	1082.668	D76N	2	1085.519	0.060837	2.305662	0.061633	5.233237	0.00165
2	10	IQRTPKIQV	7	1082.668	D76N	30	1085.689	0.0597	2.475593	0.06051	5.241644	0.005655
2	10	IQRTPKIQV	7	1082.668	D76N	120	1086.094	0.065	2.881541	0.065745	5.245813	0.003044
4	10	RTPKIQV	5	841.5254	D76N	0	841.9203	0.020499	0	0	5.238701	0.003341
4	10	RTPKIQV	5	841.5254	D76N	0.5	843.7792	0.078949	1.858954	0.081567	5.238338	0.005102
4	10	RTPKIQV	5	841.5254	D76N	1	843.7899	0.059411	1.869636	0.062848	5.233944	0.00848

4	10	RTPKIQV	5	841.5254	D76N	2	843.903	0.061814	1.982711	0.065125	5.239696	0.00559
4	10	RTPKIQV	5	841.5254	D76N	30	843.9656	0.129469	2.045349	0.131082	5.247463	0.00607
4	10	RTPKIQV	5	841.5254	D76N	120	844.3604	0.109203	2.440155	0.11111	5.248086	0.006011
6	10	PKIQV	3	584.3766	D76N	0	584.6496	0.022144	0	0	5.236691	0.005772
6	10	PKIQV	3	584.3766	D76N	0.5	585.6063	0.025537	0.956711	0.033801	5.235205	0.003586
6	10	PKIQV	3	584.3766	D76N	1	585.6784	0.01857	1.02875	0.0289	5.231026	0.004576
6	10	PKIQV	3	584.3766	D76N	2	585.706	0.029026	1.056418	0.036508	5.2424	0.003669
6	10	PKIQV	3	584.3766	D76N	30	585.8062	0.041795	1.15661	0.047298	5.241736	0.004399
6	10	PKIQV	3	584.3766	D76N	120	586.0406	0.023797	1.391005	0.032506	5.244409	0.004662
11	23	YSRHPAENGKSNF	11	1506.708	D76N	0	1507.454	0.055421	0	0	4.006848	0.004647
11	23	YSRHPAENGKSNF	11	1506.708	D76N	0.5	1510.233	0.056289	2.779584	0.078993	4.005658	0.002192
11	23	YSRHPAENGKSNF	11	1506.708	D76N	1	1510.546	0.061235	3.0928	0.08259	3.995889	0.003798
11	23	YSRHPAENGKSNF	11	1506.708	D76N	2	1510.693	0.060582	3.239417	0.082108	3.999673	0.003355
11	23	YSRHPAENGKSNF	11	1506.708	D76N	30	1510.781	0.070438	3.327904	0.089627	4.003822	0.002967
11	23	YSRHPAENGKSNF	11	1506.708	D76N	120	1510.95	0.085847	3.49672	0.102182	4.014941	0.008275
11	26	YSRHPAENGKSNFLNC	14	1836.845	D76N	0	1837.885	0.016658	0	0	5.325549	0.004138
11	26	YSRHPAENGKSNFLNC	14	1836.845	D76N	0.5	1840.368	0.062803	2.482474	0.064974	5.32088	0.003416
11	26	YSRHPAENGKSNFLNC	14	1836.845	D76N	1	1840.577	0.082147	2.691314	0.083819	5.317707	0.003015
11	26	YSRHPAENGKSNFLNC	14	1836.845	D76N	2	1840.644	0.066256	2.75877	0.068318	5.326893	0.002233
11	26	YSRHPAENGKSNFLNC	14	1836.845	D76N	30	1840.705	0.067609	2.819928	0.069631	5.325852	0.005026
11	26	YSRHPAENGKSNFLNC	14	1836.845	D76N	120	1840.848	0.089112	2.962893	0.090656	5.328826	0.004825
27	35	YVSGFHPSD	7	1008.442	D76N	0	1009	0.05294	0	0	5.155731	0.009675
27	35	YVSGFHPSD	7	1008.442	D76N	0.5	1009.853	0.075708	0.853359	0.092381	5.155433	0.002672
27	35	YVSGFHPSD	7	1008.442	D76N	1	1009.873	0.020981	0.873073	0.056946	5.15265	0.00445
27	35	YVSGFHPSD	7	1008.442	D76N	2	1009.963	0.043054	0.96329	0.068237	5.158993	0.003126
27	35	YVSGFHPSD	7	1008.442	D76N	30	1010.275	0.064609	1.275227	0.083528	5.164906	0.003668
27	35	YVSGFHPSD	7	1008.442	D76N	120	1010.533	0.055749	1.532964	0.076881	5.163911	0.003493
27	37	YVSGFHPSDIE	9	1250.569	D76N	0	1251.226	0.035702	0	0	5.890125	0.003867
27	37	YVSGFHPSDIE	9	1250.569	D76N	0.5	1251.725	0.088695	0.498272	0.095611	5.890809	0.003897
27	37	YVSGFHPSDIE	9	1250.569	D76N	1	1251.81	0.023293	0.584096	0.042628	5.888831	0.005012

27	37	YVSGFHPSDIE	9	1250.569	D76N	2	1251.8	0.084019	0.57338	0.09129	5.89572	0.003147
27	37	YVSGFHPSDIE	9	1250.569	D76N	30	1252.182	0.066804	0.955829	0.075746	5.900443	0.004558
27	37	YVSGFHPSDIE	9	1250.569	D76N	120	1252.599	0.076946	1.373162	0.084825	5.90416	0.006
27	38	YVSGFHPSDIEV	10	1349.637	D76N	0	1350.258	0.05377	0	0	6.503096	0.006615
27	38	YVSGFHPSDIEV	10	1349.637	D76N	0.5	1350.762	0.039531	0.503196	0.066738	6.508204	0.00417
27	38	YVSGFHPSDIEV	10	1349.637	D76N	1	1350.756	0.063504	0.49814	0.08321	6.505014	0.004904
27	38	YVSGFHPSDIEV	10	1349.637	D76N	2	1350.883	0.085169	0.624251	0.100723	6.511733	0.002724
27	38	YVSGFHPSDIEV	10	1349.637	D76N	30	1351.381	0.044734	1.122906	0.069945	6.510231	0.001813
27	38	YVSGFHPSDIEV	10	1349.637	D76N	120	1352.012	0.082303	1.753266	0.098311	6.515341	0.004181
29	35	SGFHPSD	5	746.3104	D76N	0	746.6234	0.025753	0	0	5.166161	0.003779
29	35	SGFHPSD	5	746.3104	D76N	0.5	747.296	0.03824	0.672525	0.046104	5.16537	0.001895
29	35	SGFHPSD	5	746.3104	D76N	1	747.3114	0.035703	0.687983	0.044022	5.167882	0.005542
29	35	SGFHPSD	5	746.3104	D76N	2	747.3486	0.028809	0.725164	0.038642	5.168905	0.006162
29	35	SGFHPSD	5	746.3104	D76N	30	747.5906	0.017758	0.967187	0.031282	5.172587	0.005092
29	35	SGFHPSD	5	746.3104	D76N	120	747.8168	0.026866	1.193349	0.037216	5.179685	0.005221
36	39	IEVD	3	475.2399	D76N	0	475.4601	0.012678	0	0	4.714399	0.004664
36	39	IEVD	3	475.2399	D76N	0.5	475.5764	0.017842	0.116327	0.021888	4.715885	0.00174
36	39	IEVD	3	475.2399	D76N	1	475.625	0.036606	0.164869	0.038739	4.712769	0.00262
36	39	IEVD	3	475.2399	D76N	2	475.6467	0.048925	0.186617	0.050541	4.713232	0.002786
36	39	IEVD	3	475.2399	D76N	30	475.7253	0.016355	0.265243	0.020694	4.7159	0.003592
36	39	IEVD	3	475.2399	D76N	120	475.8977	0.020189	0.437639	0.02384	4.716373	0.005043
36	40	IEVDL	4	588.3239	D76N	0	588.5642	0.022425	0	0	6.680618	0.002854
36	40	IEVDL	4	588.3239	D76N	0.5	588.7042	0.016148	0.139988	0.027634	6.68361	0.001128
36	40	IEVDL	4	588.3239	D76N	1	588.7118	0.017042	0.147649	0.028166	6.682476	0.002656
36	40	IEVDL	4	588.3239	D76N	2	588.742	0.011796	0.177792	0.025338	6.686014	0.002138
36	40	IEVDL	4	588.3239	D76N	30	588.9182	0.020467	0.354038	0.030361	6.687576	0.00179
36	40	IEVDL	4	588.3239	D76N	120	589.1512	0.036046	0.586998	0.042452	6.694117	0.001458
36	41	IEVDLL	5	701.408	D76N	0	701.7204	0.014448	0	0	7.563234	0.00242
36	41	IEVDLL	5	701.408	D76N	0.5	701.8782	0.013646	0.157795	0.019874	7.564886	0.002976
36	41	IEVDLL	5	701.408	D76N	1	701.8807	0.014533	0.160306	0.020493	7.565537	0.002081

36	41	IEVDLL	5	701.408	D76N	2	701.9143	0.02327	0.193884	0.027391	7.568018	0.003915
36	41	IEVDLL	5	701.408	D76N	30	702.0726	0.022971	0.352157	0.027137	7.570887	0.002899
36	41	IEVDLL	5	701.408	D76N	120	702.3062	0.027862	0.585749	0.031386	7.574921	0.004628
36	56	IEVDLLKNGERIEKVEHSDLS	20	2423.278	D76N	0	2424.558	0.05006	0	0	5.930215	0.006785
36	56	IEVDLLKNGERIEKVEHSDLS	20	2423.278	D76N	0.5	2428.695	0.115718	4.137111	0.126082	5.907037	0.006679
36	56	IEVDLLKNGERIEKVEHSDLS	20	2423.278	D76N	1	2429.072	0.055834	4.514531	0.07499	5.906008	0.00817
36	56	IEVDLLKNGERIEKVEHSDLS	20	2423.278	D76N	2	2429.462	0.12469	4.904457	0.134363	5.912385	0.007868
36	56	IEVDLLKNGERIEKVEHSDLS	20	2423.278	D76N	30	2430.25	0.084412	5.692245	0.098139	5.915541	0.009425
36	56	IEVDLLKNGERIEKVEHSDLS	20	2423.278	D76N	120	2430.889	0.126424	6.331071	0.135974	5.921006	0.007944
36	57	IEVDLLKNGERIEKVEHSDLSF	21	2570.346	D76N	0	2571.702	0.0593	0	0	6.510043	0.005635
36	57	IEVDLLKNGERIEKVEHSDLSF	21	2570.346	D76N	0.5	2575.811	0.054887	4.108488	0.080803	6.490281	0.005363
36	57	IEVDLLKNGERIEKVEHSDLSF	21	2570.346	D76N	1	2576.36	0.074025	4.658199	0.094848	6.486214	0.005025
36	57	IEVDLLKNGERIEKVEHSDLSF	21	2570.346	D76N	2	2576.811	0.102044	5.108872	0.118023	6.496608	0.006505
36	57	IEVDLLKNGERIEKVEHSDLSF	21	2570.346	D76N	30	2577.743	0.102576	6.040873	0.118484	6.49647	0.006665
36	57	IEVDLLKNGERIEKVEHSDLSF	21	2570.346	D76N	120	2578.357	0.128676	6.654776	0.141683	6.50234	0.005667
38	55	VDLLKNGERIEKVEHSDL	17	2094.119	D76N	0	2095.239	0.071616	0	0	5.66708	0.007026
38	55	VDLLKNGERIEKVEHSDL	17	2094.119	D76N	0.5	2098.962	0.075165	3.72307	0.10382	5.645787	0.011829
38	55	VDLLKNGERIEKVEHSDL	17	2094.119	D76N	1	2099.446	0.150167	4.206411	0.16637	5.641241	0.00823
38	55	VDLLKNGERIEKVEHSDL	17	2094.119	D76N	2	2099.662	0.134754	4.423029	0.152603	5.652528	0.010478
38	55	VDLLKNGERIEKVEHSDL	17	2094.119	D76N	30	2100.078	0.077455	4.838881	0.10549	5.653504	0.012226
38	55	VDLLKNGERIEKVEHSDL	17	2094.119	D76N	120	2100.54	0.118663	5.300419	0.1386	5.657938	0.010116
38	56	VDLLKNGERIEKVEHSDLS	18	2181.151	D76N	0	2182.26	0.064325	0	0	5.523705	0.006783
38	56	VDLLKNGERIEKVEHSDLS	18	2181.151	D76N	0.5	2186.342	0.084172	4.08221	0.105937	5.506265	0.006275
38	56	VDLLKNGERIEKVEHSDLS	18	2181.151	D76N	1	2186.882	0.10168	4.621338	0.120318	5.49501	0.012059
38	56	VDLLKNGERIEKVEHSDLS	18	2181.151	D76N	2	2187.242	0.183139	4.9816	0.194107	5.504729	0.010492
38	56	VDLLKNGERIEKVEHSDLS	18	2181.151	D76N	30	2187.881	0.171769	5.620985	0.183418	5.507607	0.017676
38	56	VDLLKNGERIEKVEHSDLS	18	2181.151	D76N	120	2188.302	0.085906	6.041429	0.10732	5.5105	0.00806
39	57	DLLKNGERIEKVEHSDLSF	18	2229.151	D76N	0	2230.383	0.037937	0	0	6.163867	0.002861
39	57	DLLKNGERIEKVEHSDLSF	18	2229.151	D76N	0.5	2234.443	0.121286	4.059702	0.127081	6.141096	0.00787
39	57	DLLKNGERIEKVEHSDLSF	18	2229.151	D76N	1	2235.01	0.091666	4.627199	0.099206	6.137663	0.007184

39	57	DLLKNGERIEKVEHSDLSF	18	2229.151	D76N	2	2235.45	0.165917	5.067247	0.170199	6.148499	0.00464
39	57	DLLKNGERIEKVEHSDLSF	18	2229.151	D76N	30	2236.346	0.125192	5.962842	0.130814	6.151509	0.004618
39	57	DLLKNGERIEKVEHSDLSF	18	2229.151	D76N	120	2236.717	0.105883	6.333858	0.112474	6.153202	0.005227
40	55	LLKNGERIEKVEHSDL	15	1880.024	D76N	0	1880.964	0.101283	0	0	4.988799	0.023607
40	55	LLKNGERIEKVEHSDL	15	1880.024	D76N	0.5	1884.031	0.081222	3.0674	0.129828	4.926953	0.012261
40	55	LLKNGERIEKVEHSDL	15	1880.024	D76N	1	1884.308	0.049949	3.343862	0.11293	4.922695	0.013029
40	55	LLKNGERIEKVEHSDL	15	1880.024	D76N	2	1884.568	0.111265	3.604188	0.15046	4.963348	0.006714
40	55	LLKNGERIEKVEHSDL	15	1880.024	D76N	30	1884.867	0.089303	3.903245	0.135031	4.949046	0.007262
40	55	LLKNGERIEKVEHSDL	15	1880.024	D76N	120	1885.199	0.137109	4.234661	0.170462	4.961638	0.010441
40	56	LLKNGERIEKVEHSDLS	16	1967.056	D76N	0	1968.04	0.088447	0	0	4.720812	0.006907
40	56	LLKNGERIEKVEHSDLS	16	1967.056	D76N	0.5	1971.503	0.077161	3.462583	0.117374	4.694435	0.009793
40	56	LLKNGERIEKVEHSDLS	16	1967.056	D76N	1	1971.903	0.092035	3.86254	0.127646	4.686948	0.010888
40	56	LLKNGERIEKVEHSDLS	16	1967.056	D76N	2	1972.357	0.154051	4.317127	0.177636	4.708739	0.004547
40	56	LLKNGERIEKVEHSDLS	16	1967.056	D76N	30	1972.874	0.105172	4.833741	0.13742	4.709316	0.00857
40	56	LLKNGERIEKVEHSDLS	16	1967.056	D76N	120	1973.138	0.11779	5.097649	0.1473	4.708838	0.004662
40	57	LLKNGERIEKVEHSDLSF	17	2114.124	D76N	0	2115.267	0.018129	0	0	5.64593	0.007648
40	57	LLKNGERIEKVEHSDLSF	17	2114.124	D76N	0.5	2118.899	0.020689	3.632051	0.027508	5.612994	0.002945
40	57	LLKNGERIEKVEHSDLSF	17	2114.124	D76N	1	2119.397	0.101072	4.129942	0.102685	5.612085	0.003651
40	57	LLKNGERIEKVEHSDLSF	17	2114.124	D76N	2	2119.868	0.086779	4.600904	0.088652	5.635622	0.00285
40	57	LLKNGERIEKVEHSDLSF	17	2114.124	D76N	30	2120.667	0.039189	5.400017	0.043179	5.629071	0.003873
40	57	LLKNGERIEKVEHSDLSF	17	2114.124	D76N	120	2120.933	0.023309	5.665975	0.029529	5.635914	0.004699
41	55	LKNGERIEKVEHSDL	14	1766.94	D76N	0	1767.846	0.039596	0	0	4.615409	0.012303
41	55	LKNGERIEKVEHSDL	14	1766.94	D76N	0.5	1770.768	0.103457	2.922663	0.110776	4.590341	0.006902
41	55	LKNGERIEKVEHSDL	14	1766.94	D76N	1	1771.114	0.117819	3.268153	0.124294	4.586478	0.009279
41	55	LKNGERIEKVEHSDL	14	1766.94	D76N	2	1771.33	0.115588	3.484028	0.122182	4.613358	0.003676
41	55	LKNGERIEKVEHSDL	14	1766.94	D76N	30	1771.561	0.105879	3.715206	0.113041	4.601294	0.004797
41	55	LKNGERIEKVEHSDL	14	1766.94	D76N	120	1771.718	0.124938	3.872324	0.131062	4.607164	0.010629
41	56	LKNGERIEKVEHSDLS	15	1853.972	D76N	0	1854.909	0.081775	0	0	4.422111	0.011337
41	56	LKNGERIEKVEHSDLS	15	1853.972	D76N	0.5	1858.016	0.195573	3.107049	0.211981	4.388135	0.004532
41	56	LKNGERIEKVEHSDLS	15	1853.972	D76N	1	1858.325	0.144405	3.415679	0.165951	4.38364	0.010037

41	56	LKNGERIEKVEHSDLS	15	1853.972	D76N	2	1858.545	0.218722	3.636132	0.233509	4.406762	0.007859
41	56	LKNGERIEKVEHSDLS	15	1853.972	D76N	30	1859.141	0.244435	4.231762	0.257751	4.393911	0.00948
41	56	LKNGERIEKVEHSDLS	15	1853.972	D76N	120	1859.507	0.18802	4.597997	0.205033	4.401718	0.008995
41	57	LKNGERIEKVEHSDLSF	16	2001.04	D76N	0	2002.083	0.047833	0	0	5.457772	0.009314
41	57	LKNGERIEKVEHSDLSF	16	2001.04	D76N	0.5	2005.493	0.060572	3.410709	0.077181	5.438924	0.008609
41	57	LKNGERIEKVEHSDLSF	16	2001.04	D76N	1	2005.983	0.106386	3.90058	0.116645	5.440104	0.007352
41	57	LKNGERIEKVEHSDLSF	16	2001.04	D76N	2	2006.436	0.124933	4.353459	0.133776	5.457835	0.005242
41	57	LKNGERIEKVEHSDLSF	16	2001.04	D76N	30	2007.151	0.086386	5.06864	0.098745	5.452332	0.007315
41	57	LKNGERIEKVEHSDLSF	16	2001.04	D76N	120	2007.369	0.08998	5.286243	0.101904	5.461519	0.005672
56	63	SFSKDWSF	7	1003.452	D76N	0	1003.962	0.012789	0	0	7.173949	0.003516
56	63	SFSKDWSF	7	1003.452	D76N	0.5	1005.438	0.031493	1.476267	0.033991	7.175	0.004125
56	63	SFSKDWSF	7	1003.452	D76N	1	1005.753	0.055523	1.791083	0.056977	7.173037	0.005818
56	63	SFSKDWSF	7	1003.452	D76N	2	1005.964	0.02413	2.002597	0.02731	7.18058	0.004845
56	63	SFSKDWSF	7	1003.452	D76N	30	1006.531	0.060204	2.569025	0.061548	7.178775	0.001875
56	63	SFSKDWSF	7	1003.452	D76N	120	1006.897	0.063725	2.935215	0.064996	7.183853	0.005466
57	61	FSKDW	4	682.3195	D76N	0	682.6432	0.012803	0	0	7.093418	0.011021
57	61	FSKDW	4	682.3195	D76N	0.5	683.4917	0.018397	0.848462	0.022413	7.096744	0.007216
57	61	FSKDW	4	682.3195	D76N	1	683.6225	0.030864	0.979339	0.033414	7.086856	0.011841
57	61	FSKDW	4	682.3195	D76N	2	683.7503	0.018969	1.10713	0.022885	7.101531	0.007439
57	61	FSKDW	4	682.3195	D76N	30	684.1252	0.045241	1.481968	0.047018	7.098793	0.004856
57	61	FSKDW	4	682.3195	D76N	120	684.3931	0.049184	1.749885	0.050823	7.10802	0.009605
57	63	FSKDWSF	6	916.4199	D76N	0	916.9189	0.02817	0	0	7.061413	0.008172
57	63	FSKDWSF	6	916.4199	D76N	0.5	918.0129	0.027224	1.094001	0.039175	7.061938	0.007165
57	63	FSKDWSF	6	916.4199	D76N	1	918.1934	0.041033	1.274455	0.049772	7.065255	0.005586
57	63	FSKDWSF	6	916.4199	D76N	2	918.397	0.03689	1.478071	0.046415	7.0723	0.008893
57	63	FSKDWSF	6	916.4199	D76N	30	918.9232	0.022849	2.004231	0.036272	7.076647	0.00543
57	63	FSKDWSF	6	916.4199	D76N	120	919.2836	0.061548	2.364691	0.067688	7.074162	0.007919
58	62	SKDWS	4	622.2831	D76N	0	622.4787	0.021596	0	0	6.69097	0.005184
58	62	SKDWS	4	622.2831	D76N	0.5	622.9271	0.009956	0.448412	0.023781	6.696298	0.006725
58	62	SKDWS	4	622.2831	D76N	1	623.0878	0.047898	0.609105	0.052542	6.696679	0.007405

58	62	SKDWS	4	622.2831	D76N	2	623.2	0.058414	0.721318	0.062278	6.699457	0.006737
58	62	SKDWS	4	622.2831	D76N	30	623.5651	0.070966	1.086476	0.074179	6.700865	0.008178
58	62	SKDWS	4	622.2831	D76N	120	623.6726	0.034028	1.193927	0.040303	6.710953	0.005997
58	63	SKDWSF	5	769.3515	D76N	0	769.756	0.012717	0	0	7.163129	0.008483
58	63	SKDWSF	5	769.3515	D76N	0.5	770.8771	0.021355	1.121176	0.024855	7.16137	0.005378
58	63	SKDWSF	5	769.3515	D76N	1	771.0936	0.029502	1.337596	0.032127	7.161934	0.003421
58	63	SKDWSF	5	769.3515	D76N	2	771.2671	0.035626	1.511081	0.037828	7.16652	0.005528
58	63	SKDWSF	5	769.3515	D76N	30	771.6856	0.027041	1.929587	0.029882	7.173163	0.004677
58	63	SKDWSF	5	769.3515	D76N	120	771.9722	0.030615	2.216258	0.033151	7.173267	0.008381
59	63	KDWSF	4	682.3195	D76N	0	682.6506	0.015743	0	0	6.691112	0.003426
59	63	KDWSF	4	682.3195	D76N	0.5	683.2237	0.026164	0.573116	0.030535	6.694062	0.005402
59	63	KDWSF	4	682.3195	D76N	1	683.3949	0.015845	0.744327	0.022336	6.696776	0.005515
59	63	KDWSF	4	682.3195	D76N	2	683.5409	0.014921	0.890328	0.02169	6.699512	0.005454
59	63	KDWSF	4	682.3195	D76N	30	683.9314	0.026523	1.280856	0.030843	6.700923	0.007838
59	63	KDWSF	4	682.3195	D76N	120	684.1922	0.039894	1.541622	0.042888	6.709171	0.005743
60	63	DWSF	3	554.2245	D76N	0	554.5092	0.02984	0	0	6.693405	0.004108
60	63	DWSF	3	554.2245	D76N	0.5	554.9861	0.037668	0.476901	0.048055	6.697986	0.003981
60	63	DWSF	3	554.2245	D76N	1	555.1335	0.013192	0.624273	0.032625	6.697626	0.005829
60	63	DWSF	3	554.2245	D76N	2	555.2198	0.028491	0.710599	0.041257	6.700044	0.005828
60	63	DWSF	3	554.2245	D76N	30	555.5546	0.025421	1.045422	0.0392	6.699963	0.006538
60	63	DWSF	3	554.2245	D76N	120	555.7728	0.042021	1.263621	0.051538	6.710101	0.00531
63	67	FYLLY	4	718.381	D76N	0	718.6375	0.017799	0	0	8.481759	0.003132
63	67	FYLLY	4	718.381	D76N	0.5	718.6931	0.038664	0.055553	0.042564	8.480944	0.007085
63	67	FYLLY	4	718.381	D76N	1	718.7225	0.055419	0.084981	0.058207	8.484891	0.005567
63	67	FYLLY	4	718.381	D76N	2	718.7299	0.057738	0.09235	0.060419	8.488225	0.004354
63	67	FYLLY	4	718.381	D76N	30	718.6691	0.019107	0.031537	0.026113	8.484818	0.003295
63	67	FYLLY	4	718.381	D76N	120	718.6811	0.034694	0.043559	0.038993	8.493622	0.00542
64	67	YLLY	3	571.3126	D76N	0	571.5874	0.006964	0	0	7.423967	0.001804
64	67	YLLY	3	571.3126	D76N	0.5	571.5962	0.011999	0.008771	0.013873	7.427328	0.006107
64	67	YLLY	3	571.3126	D76N	1	571.6004	0.021065	0.013004	0.022186	7.424203	0.009313

64	67	YLLY	3	571.3126	D76N	2	571.6107	0.006453	0.023346	0.009495	7.433418	0.003507
64	67	YLLY	3	571.3126	D76N	30	571.6114	0.010696	0.024028	0.012763	7.42702	0.004237
64	67	YLLY	3	571.3126	D76N	120	571.6031	0.00451	0.015694	0.008297	7.436913	0.003901
64	68	YLLYY	4	734.376	D76N	0	734.7346	0.024378	0	0	7.866215	0.002649
64	68	YLLYY	4	734.376	D76N	0.5	734.7386	0.040031	0.004009	0.046869	7.870457	0.007025
64	68	YLLYY	4	734.376	D76N	1	734.7534	0.022566	0.018846	0.033219	7.864642	0.002762
64	68	YLLYY	4	734.376	D76N	2	734.772	0.013542	0.037404	0.027887	7.874477	0.002595
64	68	YLLYY	4	734.376	D76N	30	734.76	0.012825	0.025426	0.027546	7.873372	0.002218
64	68	YLLYY	4	734.376	D76N	120	734.7844	0.036126	0.049797	0.043582	7.884034	0.002553
65	68	LLYY	3	571.3126	D76N	0	571.5365	0.026328	0	0	7.049033	0.004439
65	68	LLYY	3	571.3126	D76N	0.5	571.6277	0.036444	0.091145	0.044959	7.053975	0.00323
65	68	LLYY	3	571.3126	D76N	1	571.5956	0.050121	0.059038	0.056615	7.051413	0.005358
65	68	LLYY	3	571.3126	D76N	2	571.6405	0.063843	0.103964	0.069058	7.055225	0.00423
65	68	LLYY	3	571.3126	D76N	30	571.5994	0.08162	0.062901	0.085762	7.05155	0.005787
65	68	LLYY	3	571.3126	D76N	120	571.59	0.039028	0.05346	0.047078	7.06094	0.002345
66	71	LYYTEF	5	835.3872	D76N	0	835.7083	0.055745	0	0	7.475368	0.002762
66	71	LYYTEF	5	835.3872	D76N	0.5	835.9868	0.033393	0.278437	0.064981	7.479516	0.007897
66	71	LYYTEF	5	835.3872	D76N	1	836.036	0.003037	0.327672	0.055827	7.478558	0.002337
66	71	LYYTEF	5	835.3872	D76N	2	836.1233	0.045089	0.414912	0.071697	7.480728	0.002918
66	71	LYYTEF	5	835.3872	D76N	30	836.4304	0.089885	0.722039	0.105768	7.487556	0.005242
66	71	LYYTEF	5	835.3872	D76N	120	836.373	0.009436	0.664631	0.056538	7.497015	0.002507
67	70	YYTE	3	575.2348	D76N	0	575.518	0.012588	0	0	4.719359	0.002985
67	70	YYTE	3	575.2348	D76N	0.5	575.6553	0.023128	0.137255	0.026331	4.724251	0.003318
67	70	YYTE	3	575.2348	D76N	1	575.7339	0.02679	0.215852	0.0296	4.717884	0.004814
67	70	YYTE	3	575.2348	D76N	2	575.8456	0.013832	0.327569	0.018703	4.72082	0.004322
67	70	YYTE	3	575.2348	D76N	30	576.1787	0.023363	0.660691	0.026538	4.719228	0.003535
67	70	YYTE	3	575.2348	D76N	120	576.211	0.01874	0.693016	0.022575	4.723186	0.004813
67	71	YYTEF	4	722.3032	D76N	0	722.6583	0.013519	0	0	6.935496	0.00179
67	71	YYTEF	4	722.3032	D76N	0.5	722.8495	0.009158	0.191275	0.016329	6.93839	0.001649
67	71	YYTEF	4	722.3032	D76N	1	722.9133	0.029819	0.255018	0.032741	6.936822	0.00334

67	71	YYTEF	4	722.3032	D76N	2	723.0245	0.025919	0.366205	0.029233	6.940199	0.002477
67	71	YYTEF	4	722.3032	D76N	30	723.3216	0.021558	0.663371	0.025446	6.941348	0.001088
67	71	YYTEF	4	722.3032	D76N	120	723.3541	0.019993	0.695894	0.024134	6.947022	0.002677
67	79	YYTEFTPTEKNEY	11	1684.738	D76N	0	1685.638	0.041549	0	0	6.09402	0.003545
67	79	YYTEFTPTEKNEY	11	1684.738	D76N	0.5	1688.929	0.09563	3.290704	0.104266	6.096449	0.004767
67	79	YYTEFTPTEKNEY	11	1684.738	D76N	1	1689.102	0.059155	3.46455	0.072289	6.093189	0.000835
67	79	YYTEFTPTEKNEY	11	1684.738	D76N	2	1689.281	0.095018	3.642985	0.103705	6.099897	0.002443
67	79	YYTEFTPTEKNEY	11	1684.738	D76N	30	1689.726	0.07932	4.088413	0.089543	6.105165	0.002803
67	79	YYTEFTPTEKNEY	11	1684.738	D76N	120	1690.145	0.040076	4.50667	0.057727	6.110073	0.001897
68	71	YTEF	3	559.2399	D76N	0	559.512	0.015423	0	0	6.315063	0.002264
68	71	YTEF	3	559.2399	D76N	0.5	559.6779	0.00893	0.165876	0.017822	6.318441	0.003122
68	71	YTEF	3	559.2399	D76N	1	559.7463	0.012575	0.234261	0.0199	6.318147	0.004968
68	71	YTEF	3	559.2399	D76N	2	559.8185	0.015949	0.30643	0.022186	6.320369	0.001758
68	71	YTEF	3	559.2399	D76N	30	560.0993	0.011126	0.587266	0.019017	6.32375	0.005123
68	71	YTEF	3	559.2399	D76N	120	560.131	0.01237	0.618961	0.019771	6.327993	0.002371
68	78	YTEFTPTEKNE	9	1358.611	D76N	0	1359.335	0.022708	0	0	5.117421	0.001906
68	78	YTEFTPTEKNE	9	1358.611	D76N	0.5	1362.578	0.029708	3.242787	0.037393	5.113891	0.001197
68	78	YTEFTPTEKNE	9	1358.611	D76N	1	1362.817	0.036409	3.482124	0.04291	5.112545	0.003549
68	78	YTEFTPTEKNE	9	1358.611	D76N	2	1362.84	0.048997	3.505111	0.054003	5.119068	0.004283
68	78	YTEFTPTEKNE	9	1358.611	D76N	30	1362.924	0.023224	3.588972	0.032481	5.118168	0.001543
68	78	YTEFTPTEKNE	9	1358.611	D76N	120	1363.155	0.040479	3.819869	0.046413	5.125546	0.00351
68	80	YTEFTPTEKNEYA	11	1592.712	D76N	0	1593.552	0.028408	0	0	5.600869	0.006041
68	80	YTEFTPTEKNEYA	11	1592.712	D76N	0.5	1596.775	0.062831	3.222343	0.068954	5.603039	0.003614
68	80	YTEFTPTEKNEYA	11	1592.712	D76N	1	1597.015	0.030227	3.462511	0.041481	5.603238	0.004739
68	80	YTEFTPTEKNEYA	11	1592.712	D76N	2	1597.072	0.083223	3.519458	0.087938	5.607384	0.002116
68	80	YTEFTPTEKNEYA	11	1592.712	D76N	30	1597.539	0.063462	3.986581	0.06953	5.612976	0.00385
68	80	YTEFTPTEKNEYA	11	1592.712	D76N	120	1597.845	0.026842	4.293044	0.039083	5.61474	0.001119
68	81	YTEFTPTEKNEYAC	12	1695.721	D76N	0	1696.691	0.033301	0	0	5.829382	0.002009
68	81	YTEFTPTEKNEYAC	12	1695.721	D76N	0.5	1699.946	0.01535	3.255367	0.036668	5.831265	0.002579
68	81	YTEFTPTEKNEYAC	12	1695.721	D76N	1	1700.209	0.046802	3.517898	0.05744	5.832393	0.004052

68	81	YTEFTPTEKNEYAC	12	1695.721	D76N	2	1700.397	0.054762	3.705859	0.064092	5.836649	0.002803
68	81	YTEFTPTEKNEYAC	12	1695.721	D76N	30	1700.811	0.112606	4.119831	0.117427	5.839717	0.002309
68	81	YTEFTPTEKNEYAC	12	1695.721	D76N	120	1701.029	0.062776	4.338218	0.071062	5.843348	0.003584
69	78	TEFTPTEKNE	8	1195.548	D76N	0	1196.053	0.038487	0	0	4.633931	0.001797
69	78	TEFTPTEKNE	8	1195.548	D76N	0.5	1199.378	0.07173	3.324956	0.081403	4.628378	0.001263
69	78	TEFTPTEKNE	8	1195.548	D76N	1	1199.512	0.13417	3.459253	0.139581	4.62642	0.002086
69	78	TEFTPTEKNE	8	1195.548	D76N	2	1199.588	0.081221	3.53473	0.089878	4.631598	0.00084
69	78	TEFTPTEKNE	8	1195.548	D76N	30	1199.462	0.119278	3.408791	0.125333	4.631053	0.0031
69	78	TEFTPTEKNE	8	1195.548	D76N	120	1199.502	0.074113	3.448845	0.08351	4.63771	0.004475
69	80	TEFTPTEKNEYA	10	1429.648	D76N	0	1430.283	0.131149	0	0	5.249251	0.007071
69	80	TEFTPTEKNEYA	10	1429.648	D76N	0.5	1433.509	0.090391	3.226033	0.159281	5.246223	0.004396
69	80	TEFTPTEKNEYA	10	1429.648	D76N	1	1433.616	0.09163	3.332767	0.159988	5.24679	0.00493
69	80	TEFTPTEKNEYA	10	1429.648	D76N	2	1433.762	0.137737	3.478928	0.190188	5.249783	0.004555
69	80	TEFTPTEKNEYA	10	1429.648	D76N	30	1434.004	0.263598	3.7207	0.294421	5.256145	0.004606
69	80	TEFTPTEKNEYA	10	1429.648	D76N	120	1434.223	0.103991	3.93952	0.167374	5.254674	0.005372
69	81	TEFTPTEKNEYAC	11	1532.657	D76N	0	1533.423	0.050128	0	0	5.511961	0.003575
69	81	TEFTPTEKNEYAC	11	1532.657	D76N	0.5	1536.674	0.087762	3.250638	0.101069	5.513573	0.002595
69	81	TEFTPTEKNEYAC	11	1532.657	D76N	1	1536.786	0.068081	3.36327	0.084545	5.512677	0.003953
69	81	TEFTPTEKNEYAC	11	1532.657	D76N	2	1536.975	0.077953	3.551317	0.09268	5.520573	0.00395
69	81	TEFTPTEKNEYAC	11	1532.657	D76N	30	1537.201	0.115381	3.77734	0.1258	5.520671	0.00464
69	81	TEFTPTEKNEYAC	11	1532.657	D76N	120	1537.442	0.069314	4.018915	0.085541	5.524379	0.003602
82	100	RVNHVTLSQPKIVKWDRDM	17	2322.25	D76N	0	2323.557	0.121452	0	0	5.440278	0.006468
82	100	RVNHVTLSQPKIVKWDRDM	17	2322.25	D76N	0.5	2325.908	0.103156	2.351023	0.159348	5.433992	0.004909
82	100	RVNHVTLSQPKIVKWDRDM	17	2322.25	D76N	1	2326.31	0.155154	2.75376	0.197037	5.432372	0.00908
82	100	RVNHVTLSQPKIVKWDRDM	17	2322.25	D76N	2	2326.802	0.080088	3.245874	0.145481	5.447464	0.009645
82	100	RVNHVTLSQPKIVKWDRDM	17	2322.25	D76N	30	2327.814	0.082409	4.257821	0.146772	5.446473	0.00519
82	100	RVNHVTLSQPKIVKWDRDM	17	2322.25	D76N	120	2328.239	0.077003	4.682701	0.143806	5.453467	0.005137
91	100	PKIVKWDRDM	8	1287.688	D76N	0	1288.324	0.060703	0	0	5.392919	0.008206
91	100	PKIVKWDRDM	8	1287.688	D76N	0.5	1289.235	0.016884	0.910159	0.063007	5.389257	0.010195
91	100	PKIVKWDRDM	8	1287.688	D76N	1	1289.508	0.09171	1.183898	0.10998	5.387402	0.011697

91	100	PKIVKWDRDM	8	1287.688	D76N	2	1289.717	0.025919	1.392436	0.066005	5.393899	0.004713
91	100	PKIVKWDRDM	8	1287.688	D76N	30	1290.165	0.08011	1.840545	0.100511	5.400335	0.009265
91	100	PKIVKWDRDM	8	1287.688	D76N	120	1290.344	0.108167	2.019605	0.124036	5.406564	0.011296
94	100	VKWDRDM	6	949.456	D76N	0	949.9472	0.028244	0	0	5.376492	0.006371
94	100	VKWDRDM	6	949.456	D76N	0.5	950.8844	0.039736	0.937191	0.048751	5.373985	0.003686
94	100	VKWDRDM	6	949.456	D76N	1	951.1041	0.04166	1.156906	0.050332	5.373646	0.00532
94	100	VKWDRDM	6	949.456	D76N	2	951.2182	0.034475	1.271019	0.044567	5.378272	0.002216
94	100	VKWDRDM	6	949.456	D76N	30	951.5023	0.052068	1.555096	0.059235	5.3854	0.006145
94	100	VKWDRDM	6	949.456	D76N	120	951.7018	0.104875	1.754623	0.108611	5.389125	0.006639
95	100	KWDRDM	5	850.3876	D76N	0	850.9415	0.035203	0	0	5.385709	0.01017
95	100	KWDRDM	5	850.3876	D76N	0.5	851.7967	0.016613	0.8552	0.038926	5.383661	0.006907
95	100	KWDRDM	5	850.3876	D76N	1	852.0077	0.019531	1.066275	0.040258	5.380366	0.006428
95	100	KWDRDM	5	850.3876	D76N	2	852.1425	0.027319	1.201077	0.04456	5.385971	0.006424
95	100	KWDRDM	5	850.3876	D76N	30	852.4098	0.044484	1.468365	0.056728	5.394183	0.009997
95	100	KWDRDM	5	850.3876	D76N	120	852.5296	0.085444	1.588183	0.092412	5.393588	0.009059

References

[1] Cornwell, O., Radford, S.E., Ashcroft, A.E., Ault, J.R.: Comparing Hydrogen Deuterium Exchange and Fast Photochemical Oxidation of Proteins: a Structural Characterisation of Wild-Type and ΔN6 β_2-Microglobulin. J. Am. Soc. Mass Spectrom., 29, 2413-2426, 2018

[2] Bush, M F., Hall, Z., Giles, K., Hoyes, J., Robinson, C.V., Ruotolo, B.T.: Collision Cross Sections of Proteins and Their Complexes: A Calibration Framework and Database for Gas-Phase Structural Biology. Anal. Chem., 82, 9557-9565, 2010
